# Cosmopolitanism in the depths of Barbaricum evidenced by archaeogenomic data from the Late Iron Age Goth community of the Masłomęcz group

**DOI:** 10.1186/s13059-026-03969-4

**Published:** 2026-01-28

**Authors:** Michał Golubiński, Mateusz Baca, Danijela Popović, Leo Speidel, Stephan Schiffels, Barbara Niezabitowska-Wiśniewska, Andrzej Kokowski, Martyna Molak

**Affiliations:** 1 Centre of New Technologies, University of Warsaw, ul. S. Banacha 2C, Warsaw, 02-097 Poland; 2 Center for Interdisciplinary Theoretical and Mathematical Sciences (iTHEMS), RIKEN, 2-1 Hirosawa, Wako, Saitama 351-0198 Japan; 3 Max Planck Institute for Evolutionary Anthropology, Deutscher Pl. 6, Leipzig, 04103 Germany; 4 Institute of Archaeology, Faculty of History and Archaeology, Maria Curie-Skłodowska University, Plac Marii Curie Skłodowskiej 4, Lublin, 20–031 Poland

**Keywords:** Masłomęcz group, Goths, Population genomics, Long-range mobility

## Abstract

**Background:**

High mobility and extensive trade and military interactions are well recognized throughout the Late Iron Age Europe. The extremely rich archaeological record for the Masłomęcz group – a Goth-associated assemblage flourishing between 2^nd^ and 4^th^ century CE in what is now eastern Poland – has long been providing evidence for their wide cross-cultural contacts. However, the extent to which these were ephemeral or involved long-term immigration and interbreeding, remained unresolved.

**Results:**

Here, by obtaining archaeogenomic data from 37 burials and reanalysing published data, we provide evidence that, while the Masłomęcz group was built mostly on Scandinavian-derived ancestry it extensively assimilated individuals from diverse directions and distances, including the Baltics, the Balkans and even further into the Mediterranean, creating a highly genetically heterogenous population. Additionally, we shed more light on the burial customs of this community by finding no close kin relations within multiperson burials.

**Conclusions:**

Our findings provide evidence for long-range mobility far outside the borders of the Roman Empire. The Masłomęcz group was a highly open community embracing external contacts and immigration, perhaps contradicting popular presumptions about the so-called barbarians.

**Supplementary Information:**

The online version contains supplementary material available at 10.1186/s13059-026-03969-4.

## Background

**Fig. 1:**
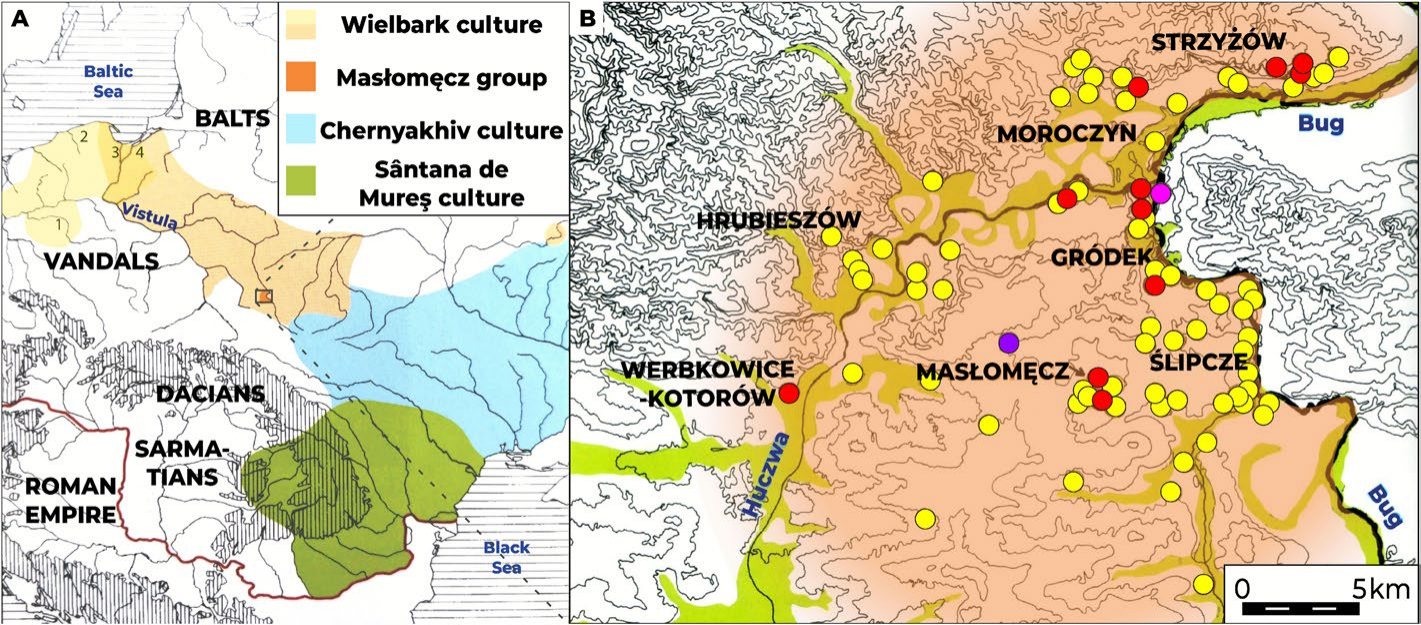
Geographic and culture context for the Masłomęcz Group. **A** The range of Wielbark culture (approximate maximum range in periods: between the 2^nd^ and mid-3^rd^ cent. CE – light cream colour; late 3^rd^ – 4^th^ cent. CE – dark cream) and other Goth complex cultures, i.e. Chernyakhiv (light blue) and Sântana de Mureş (green). Masłomęcz group marked with dark orange. Wielbark archaeological sites with published genomic data were labelled with numbers: 1 – Kowalewko, 2 – Czarnówko, 3 – Pruszcz Gdański, and 4 – Weklice. **B** Zoom in on the range of Masłomęcz Group archaeological complex along the Hrubieszów Basin (coloured lime-green) with major archaeological sites labelled and the findings of traces of the culture marked with filled circles (yellow – settlements, red – cemeteries, purple – treasure of gold coins, pink – sword found in the Bug River)

The history of the association of Germanic tribes known as Goths has been extensively described in historical [1] and archaeological [2] literatures. Despite the ongoing debate on the exact place of their origin, it has been established that the spread of this community commenced in the Baltic Sea area and followed a south-easterly direction towards the Black Sea [3, 4]. Throughout this traverse, which took place between the 1^st^ and the 4^th^ century CE, they were coming into contact with a vast diversity of tribes inhabiting the newly conquered territories, including other Germanic tribes, such as Vandals, in what is now southern Poland, as well as other ethnic groups in the territories of today's Ukraine and the Balkans [4], such as the Sarmatian nomads representing the Iranian linguistic group, Dacian and Thracian tribes, as well as the multi-ethnic community of Greek and Roman cities in the Black Sea zone. These contacts left pronounced imprints on the culture of the Gothic communities, which, along with the geographical and, hence, political distance growing between them, led to diversification within the Goth cultural circle into archaeologically distinct groups [3]: the Wielbark (1^st^ to mid-5^th^ century), Chernyakhiv (mid-3^rd^ to mid-5^th^ century), and Sântana de Mureş (late 3^rd^−5^th^ century) cultures and the Masłomęcz group (late 2^nd^ to mid-5^th^ century, distinguished within the Wielbark culture) (Fig. 1A). The polycultural, and, arguably, polyethnic, nature of these people, which started at latest with the Wielbark culture, was recognized at the end of the 1980s [5]. Excavations and examination of the cemeteries of the Masłomęcz group have strengthened this hypothesis even further [6].

The group of Goths that settled in the eastern part of the Hrubieszów Basin (currently central part of Poland’s eastern border) is referred to as the Masłomęcz group, after the major archaeological site associated with these people located in the present village of Masłomęcz, 50 km East of Zamość [7, 8]. The presence of Goths in this region has been marked to date by identified traces of ca. 70 settlements and 10 cemeteries as well as notable isolated finds such as the Metelin treasure [9] or a sword found in the Bug River near Gródek [10] and a large collection of metal artefacts, mainly ornaments and elements of attire, from several dozen sites of the Masłomęcz group [11] (Fig. 1B).

The Masłomęcz group is notable for the exceptional richness in its archaeological material culture. Over 800 graves have been excavated thus far; the vast majority come from the two largest cemeteries at sites Masłomęcz 15 and Gródek 1C. The wealth of grave inventories makes it possible to accurately date individual burials, and the excellent natural conditions in which the bones were deposited have resulted in their very good preservation. They are thus excellent material for extensive anthropological analyses.

The Masłomęcz group population also presents a wide diversity of burial rites, which can be used as a starting point to linking genetic and social ties among the individuals. The rites included multiple reopening of the graves, body fragmentation and only partial deposition of the deceased in the grave, as well as multiperson burials. In some graves, the main burial is accompanied by detached bones, sometimes skulls, of other individuals. In addition, single bones of adults were sometimes deposited in children's graves. Skeletons of even very young children have been remarkably preserved. Moreover, burial rituals and furnishing of some of the Masłomęcz group graves suggest affiliation with Sarmatian [12, 13], Baltic [10, 12, 14] and Sântana de Mureş [15, 16] cultures among its members.

Despite the immensity of archaeological, historical and anthropological inquiry, there is still a considerable number of questions that could not be addressed within the frameworks of the respective disciplines. The extent and exact source of the incoming Scandinavian ancestry, the extent and composition of the local preceding Oksywie Culture ancestry in the formation of the Wielbark Cultural Complex as well as the particular Wielbark group which was a source for the Gothic expansion towards the Hrubieszów Basin are among these questions. Furthermore, the level to which the presence of the myriad of external cultural artefacts and customs found among the Masłomęcz Group burials is reflected by non-typically-local biological affinities of the buried people has not yet been fully addressed by the hitherto applied methodology.

The recent developments in genomic methods, and particularly ancient DNA analysis, present an opportunity to further supplement the existing knowledge about the origin and history of the Masłomęcz group. Moreover, thanks to the diversity of their customs and practices and the richness of well-preserved skeletal material, this population provides an unusual potential for large-scale, comprehensive, fine-grained, multidisciplinary investigation. This, in turn, is expected not only to significantly increase our knowledge about the Masłomęcz group community itself and even about the Goths themselves. It should also provide valuable insight into the organization and structure of ancient societies from outside the well-described Greco-Roman cultural circle and give us a glimpse of the worldview, values and individual connections of the so-called “barbarians”.

The Masłomęcz group as well as the other Goth populations have already been the subjects of archaeogenetic research. A study of mitochondrial DNA variation in the Masłomęcz group by Stolarek et al. [17] inferred ties of this group to, on the one hand, other Wielbark and Scandinavian populations, and on the other to the Chernyakhiv group from the Pontic Steppe. Genome-wide data for several individuals representing Masłomęcz Group as well as three sites in the Northwestern part of the Wielbark complex range (Kowalewko, Pruszcz Gdański and Czarnówko; Fig. 1A) published by the same group [18] investigated the continuity and affiliations of these populations. However, this study mostly looked at the Iron Age group as a whole and did not focus on the characteristics of the particular sites and archaeological complexes within. Moreover, their interpretation of the obtained results has raised substantial controversy and has been challenged by subsequent literature, which pointed out archaeological inconsistencies [6, 19] and rejected the conclusion of long-term genetic continuity in the region [20] as postulated by Stolarek et al. [18]. Among the Gothic groups, genomic data for Weklice Wielbark site (Fig. 1A) were also studied [21] but only as a part of a larger transect study with no particular focus on the group itself.

Therefore, despite the availability of genomic data, there is much room for investigation into the genetic make-up and affinities of the Goth cultural complex groups, and the Masłomęcz group in particular. In this study, we performed genomic analyses on 37 individuals representing the largest Masłomęcz group necropoles at Masłomęcz 15 and Gródek 1C as well as three smaller burial sites of Moroczyn 25, Strzyżów and Werbkowice Kotorów II (Additional file 1: Supplementary Text 1, Additional file 1: Fig. S1-S4). We aimed at exploring the internal structure of the Masłomęcz group population, including the relations between the burial context, furnishings and location and the biological ancestry of the buried persons as well as the familial relationships among them. We also explored the changes in this structure throughout the duration of the Masłomęcz group settlement in the Hrubieszów Basin, and evaluated the connections of the Masłomęcz group to other populations from the Goth cultural complex and beyond it as observable at the level of biological ancestry of the buried individuals.

## Results

### Sequencing and authentication

Skeletal samples from 43 individuals were collected for genetic analysis and processed at the Laboratory of Paleogenetics and Conservation Genetics of the University of Warsaw (Additional file 2: Table S1) as a part of a larger project and as a pilot investigation of the Masłomęcz group genomic structure. Initial screening involved DNA extraction and double-stranded library preparation for two independent aliquots of bone or tooth powder per individual. The libraries were shotgun-sequenced targeting ca. 1 million reads to assess preservation and, whenever possible, contamination level and genetic sex. Endogenous DNA content, defined as a ratio of the number of unique reads mapping to the reference to the overall number of raw reads, exceeded 5% in 36 individuals and varied from ~0% to 52% (median: 13%; Additional file 2: Table S2). As a result of the applied quality control, one sample (PL057) was excluded from further analyses as being potentially contaminated (Additional file 1: Supplementary Text 2, Additional file 2: Table S3, Additional file 1: Fig. S5). Five further samples yielded few genetic data which precluded in-depth analyses, although providing sufficient information for sex, Y chromosome haplogroup and mitochondrial haplogroup determination for four, two and one of these individuals, respectively. The remaining 37 individuals provided sufficient data for comprehensive genomic analyses with 29 of these having sufficient coverage for inclusion in the imputation-reliant Identity By Descent (IBD) analyses.

We determined genetic sex for 41 individuals and found that 24 individuals were females and 17 were males. For most of the individuals for which osteological sex assignment was available, genetic sex was concordant with this assignment. Two individuals assigned osteologically as juvenile females (PL066 and PL067) turned out to be genetic males and the presumed adult male PL072 was genetically female.

We merged our newly obtained genomic data with those for individuals from the Masłomęcz 15 archaeological site published by Stolarek et al. [18] (Additional file 2: Table S4). The merged Masłomęcz group population, hereafter MSL, comprised 50 individuals (37 newly sequenced – each labelled with “PL” prefix and a number, and 13 from Stolarek et al. – each labelled with “PCA” prefix and a number) with sufficient data (minimum 20k SNPs) for inclusion in population genomic and kinship analyses. Twenty of these individuals (40%) were genetically determined as males and 30 as females (χ^2^ test for equal sex ratio’s *p*-value = 0.16). Of these, 31 of the MSL individuals could be subjected to IBD analyses.

### Uniparentally inherited markers

Among the 38 newly sequenced individuals, for which it was possible to determine mitochondrial haplogroups (average mitochondrial genome coverage between 10 and 226×; median 75×) there were at least 30 different mitochondrial lineages. Macrohaplogroups H, U and nonH-HV were the most prevalent and accompanied by other haplogroups, all representing known European diversity, at lower frequencies (Additional file 2: Table S5). Comparably high levels of mitochondrial diversity can be observed in other Wielbark groups [18]. The mitochondrial haplogroup distribution in the MSL population did not reflect that of modern nor of the Bronze Age population from the region very closely but the overall haplogroup composition between these periods was similar. Yet, a formal statistical comparison of haplogroup frequencies was precluded by the low sample size.

We determined Y chromosome haplogroups for all the 17 genetically male individuals (Additional file 2: Table S6). Twelve males (71%) belonged to the I1 haplogroup, four (24%) to R1a and one to J2b. Haplogroup I1 currently occurs mainly among the modern populations living in Scandinavia and has been present in Scandinavian populations at least since the early Bronze Age [22, 23], although it may have originated elsewhere. Eight individuals represent haplogroup I-Y2245, two I-FGC41265, one I-Z2041 (xZ2040) and one I-Y46812.

Individual PL067, belonging to haplogroup R1a, represents a subclade (Z284) found almost exclusively in modern Scandinavian populations [24, 25] and ancient samples from Scandinavia and from a Viking context [26]. Three individuals belong to the R-Z280 subclade. Among them, individual PL046 from Strzyżów belonged to subclade R-YP6213, which is virtually absent from most modern populations, except for samples from Northwestern Europe (present-day England [27]). Individuals PL052 from Gródek and PL066 from Masłomęcz represent the R-CTS1211 branch. The majority of its subclades, apart from single branches found in northern or western European populations [28], are deeply rooted in contemporary populations of Slavic and Baltic settlement origins. Individual PL085 represents haplogroup J2b (branch L283), which is most commonly found among contemporary individuals from the Balkans [27, 29]. Additionally, among the five males from Stolarek et al. [18], three also belonged to I1 haplogroup, one to N-VL29 haplogroup present among Scandinavian and Baltic populations, and one to E1b haplogroup.

Altogether in the MSL group, based on the Y-chromosome haplogroups, the ancestry of: 17 individuals (77%) can be linked to the Baltic Sea basin populations with the closest affinities to Scandinavians; two individuals (9%) with contemporary broadly Balto-Slavic groups, next two (9%) remain ambiguous regarding their precise biogeographic origins and one individual shows the closest affinity to populations from the Balkan Peninsula. The 22 male individuals represent a minimum of 16 separate lineages. This indicates high diversity of the male lineages in the MSL group.

### Kinship analysis

We estimated kinship using READv2 [30], BREADR [31], NGSRelate [32] for all pairs of MSL individuals and using ancIBD [33] among the 31 MSL individuals with a minimum of 500k SNPs genotyped (Additional file 2: Table S7). We detected close relatedness between individuals PL076 and PCA0105 (Additional file 1: Fig. S6-S8). READv2 and BREADR classified them as first-degree relatives, and NGSRelate as half-siblings. Individual PCA0105 was thus removed from all analyses sensitive to relatedness of included individuals (*f*_3_ MDS, *f*_3_ NJ tree). Unfortunately, this pair could not be included in the ancIBD analysis due to the insufficient number of SNPs genotyped for PCA0105.

Forty-two pairs of individuals were classified as third-degree relatives by READv2. Two of these pairs were included in ancIBD analysis [33]. It detected no IBD tracts for pair PL048 – PL076, and one 12 cM IBD tract for pair PL048 – PL070. The number and length of the IBD segments called is hence too low for more precise inference of relatedness between the individuals. Among the other third-degree relations detected by READv2, a noteworthy case included three females from grave 20 in Masłomęcz among which third-degree relationships were detected between PL040 and PL042 and between PL041 and PL042 (while no such relatedness signal was detected between PL040 and PL041). All three women have different mitochondrial haplogroups thus are not related matrilineally. Moreover, PL042 was included in 17 third-degree relations detected by READv2. Apart from PL042, other highly connected individuals included PL049 male, who appeared in 10 third-degree related pairs, and PL071 male in nine.

IBD tracts (at least 2 tracts of min. 12 cM) were only detected in four pairs of individuals (PL067 – PL083, PL062 – PL063, PL060 – PL063, and PL054 – PL060) out of 465 analysed pairs among the 31 individuals eligible for IBD analysis. Each of these points to a relationship no closer than fourth degree. One of the pairs linked individuals from different cemeteries (PL054 from Gródek 1C and PL060 from Masłomęcz 15) while the other three were within the Masłomęcz 15 cemetery.

Runs of homozygosity (ROH) analysis using hapROH [34] showed rather low levels of consanguinity among the parents of the studied MSL individuals (31 individuals had sufficient amount of data for this analysis). This result points to a high effective population size of the community and no custom of reproduction among close kin individuals (Additional file 2: Table S8, Additional file 1: Fig. S9). Individual PL083 (male) is a clear outlier with six long (> 12 cM) ROH stretches detected, which suggests close kin relations (first or second-cousin degree) between his parents. Elevated ROH presence was also detected in PL046 (also male) which suggests a second-cousin relation between his parents.

### Population genomics

**Fig. 2:**
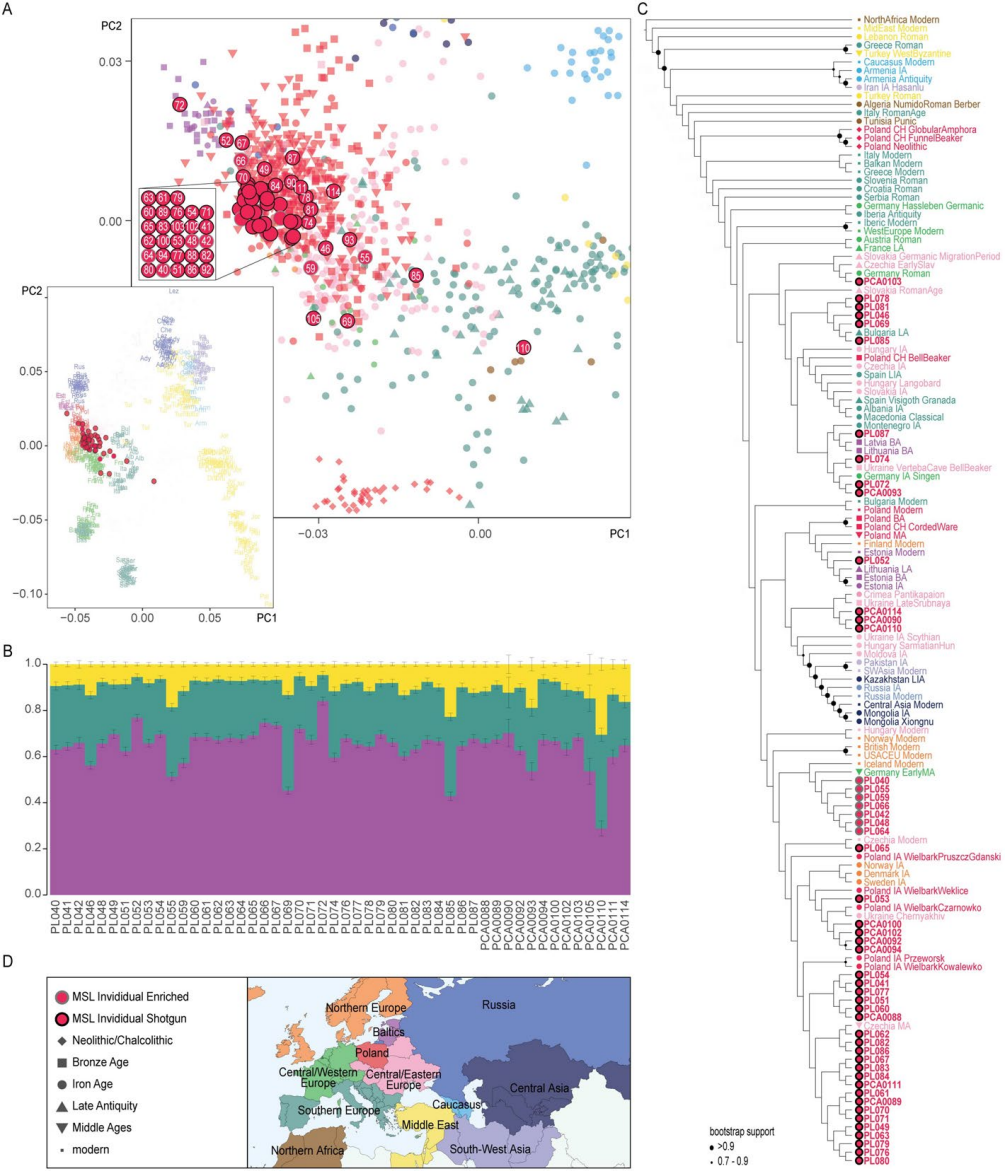
Genetic affinities of the MSL individuals. **A** PCA of the allele frequencies of the Human Origins SNP set estimated using West Eurasian modern human data [35] with ancient individuals projected. On the left – full PCA plot showing the modern data and the projected MSL samples. On the right – a zoom in of the PCA to the portion of relevance for the studied samples. Here, the modern samples were removed for readability, and the labelled projected MSL samples were shown together with Iron Age and Late Antiquity genomes from relevant populations and all ancient genomes from Poland (which, for readability, were not included in the full plot to the left). Each point represents an individual. MSL individual labels were shortened for visibility to the two or three terminal digits. For the portion of the graph where the labels of the MSL samples overlapped, the labels were removed from the markers and instead singled out in a box on the left side with the position within the box roughly corresponding to the position within the cluster. **B** Unsupervised Admixture analysis with all samples from Poland projected onto the components estimated using West Eurasian modern human data [35] (200 bootstrap iterations) for K = 3 (lowest CV error among the tested K (2 to 8). Only the components for the MSL samples are shown (full results provided in Additional file 2: Table S10). Colours of the components correspond to the regions in which the given component appears maximized among the modern-day populations according to the colour legend provided in panel D. **C** Neighbour-joining cladogram for the outgroup *f*_3_-statistics [1-*f*_3_(population1/individual1, population2/individual2; Yoruba)] matrix. Note that the branch lengths are not representative of genetic distance. Nodes with bootstrap support values above 0.7 were marked with black circles. **D** Colour and marker legend for Fig. 2A-C. The map shows regional groupings and colour-scheme assigned to the individuals’ skeletal remains’ countries of origin according to modern-day political division

Principal Component Analysis (PCA) comprising wide range of modern and ancient West Eurasian populations (Fig. 2A, Additional file 2: Table S9) placed most of the MSL individuals within the cluster including other individuals from Poland and the rest of the Iron Age and Late Antiquity Central and Northern Europe. One individual – PCA0110, is notably shifted towards the southern populations (result also obtained, but not discussed, in the original publication, [18]). There was also a noteworthy (although not nearly as pronounced as in PCA0110) shift of PL085, PL055, PCA093 and PL046 individuals towards South-European groups and PL069, PCA0105, and PL059 towards Southern and Western European groups in the PCA. Another group of individuals (PL072 and less so PL052, PL067 and PL066) were shifted towards the Baltic populations.

Unsupervised Admixture analysis showed rather consistent genomic composition among the MSL individuals (Fig. 2B) and, generally, similarity of the pattern to other post-Neolithic populations from Central Europe (Additional file 2: Table S10). The lowest CV error was estimated at K = 3 (Additional file 2: Table S10A). The ancestry composition variation among the MSL individuals was not very strongly pronounced. Some individuals, however, displayed a somewhat distinct pattern. PL046, PL055, PL059, PL069, PL074, PL085, PCA0093, PCA0105 and PCA0110 had more of component 2 and 3 in K = 3 which was maximized in, respectively, modern Sardinian and Yemenite Jew populations, compared to the remaining individuals. By contrast, PL052 and PL072 were depleted in these components and had increased proportions of component 1 in K = 3, which is maximized in modern Estonian and Russian populations.

Outgroup *f*_3_(Ind/Pop1, Ind/Pop2; Yoruba) statistics (Additional file 2: Table S11) were used to construct a neighbour-joining cladogram (in form of 1-*f*_3_ values; Fig. 2C). Here, most of the MSL samples clustered in a clade together with other Polish Wielbark and Przeworsk, Ukraine Chernyakhiv, Scandinavian Iron Age as well as Medieval and modern Czech groups. Sister to this clade laid a clade comprising seven MSL individuals, Early Medieval German group and modern Iceland. We investigated whether the distinctiveness of this clade could have been caused by any artefacts of laboratory and bioinformatic processing, which revealed that all the MSL samples within this clade, and none of the MSL samples outside it, had their genetic data obtained predominantly using the Human Affinities Prime Plus panel (Arbor Biosciences) target enrichment. Interestingly, the Early Medieval Germany and modern Iceland data with which they clustered came from shotgun sequencing. Our extensive tests for the effects of this bias, previously described by Davison et al. 2023 [36], on the obtained results (Additional file 1: Supplementary Text 3, Additional file 2: Table S12, Additional file 1: Fig. S10-S13) showed that, while it causes consistent clustering of the samples depending on the sequencing strategy within the MSL group, the bias does not seem to substantially affect the affinity of the MSL group nor of the particular MSL individuals towards external groups.

Returning to the outgroup *f*_3_ neighbour-joining cladogram analysis, the MSL samples laying outside the clade comprising most MSL individuals were scattered throughout the tree, grouping with Steppe and Central Asian groups (PCA0090, PCA0110 and PCA0114), with Baltic and Central/Eastern European groups (PL052, PL072, PL074, PL087, PCA0093 and PCA0103), with Southern European groups (PL046, PL069, PL078, PL081 and PL085). However, the robustness of the tree topology was notably very weak and most groupings were very unstable as shown by bootstrap analysis showcasing that the differences in *f* statistics between the analysed groups are probably largely driven by a very small proportion of SNPs.

Similar to the PCA plot, Multidimensional Scaling (MDS) plot based on the same outgroup *f*_3_ statistics in form of (1 – *f*_3_) values, placed the MSL individuals together with other Central as well as Northern European individuals (Additional file 1: Fig. S14). Likewise, sample PCA0110 showed strong affinity with Mediterranean samples, with PL085, PL069 and PL046 less so, respectively.

Outgroup *f*_3_ affinities maps for each MSL individual (Additional file 1: Fig. S15) further corroborated the heterogeneity of ancestry shared with reference populations. Most of the populations sharing maximum ancestry with the MSL individuals were from either Central Europe, Eastern Baltic Region or Scandinavia, usually from the Iron Age, although the outlier individuals show the highest affinity to populations from the Balkans (PL046, PL069, PL085), Bronze Age Ukraine (PL074, PL077, PCA0110), and Western Europe (PCA0093).

Admixture *f*_3_ statistics for MSL individuals in form *f*_3_(Test.Pop1, Test.Pop2; MSL.Ind) yielded no significant negative values, thus providing no statistical support for any individual being admixed of either of the test population pairs (Additional file 2: Table S13).

Homogeneity of the MSL was tested using *f*_4_ statistics in form *f*_4_(Yoruba, Test.Pop; MSL.Ind1, MSL.Ind2). The considerable number (for 2686 out of 111 993 triplets; 2.4%) of values with |Z|> 3 indicated that MSL individuals are differentially related to the test populations, i.e. the MSL is not genetically homogenous (Additional file 2: Table S14). The fraction of pairs of individuals exhibiting differential levels of affinities to the test populations was higher than in the other four Wielbark populations (0.15–1.04%, all χ^2^ test *p*-values < 10^–3^; Additional file 2: Table S14F). The presence of outliers was further investigated using *f*_4_ statistics in the form *f*_4_(MSL.Pop, Test.Pop; MSL.Ind, Yoruba). In this case no MSL individual was, however, significantly closer to either of the tested 87 other populations than to the MSL population (i.e. its source population; Additional file 2: Table S15). This included the genetic outliers PL085 and PL069 who were significantly closer to the MSL population than to either of the other test population, and PCA105 and PCA110, whose affinity could not be discerned between the MSL population and only a handful of test populations (all from Iron Age to Medieval Balkans, Baltics and Central and Eastern Europe) while being significantly closer to the MSL population against all the other test populations.

Affinities of the MSL individuals, and MSL population as a whole, to selected populations were further tested using *f*_4_(Test.Pop1, Test.Pop2; MSL.Pop/MSL.Ind, Yoruba) (Additional file 2: Table S16). Populations that were most often significantly closer to MSL individuals than other populations were (decreasing, from the most frequently closer to MSL): Wielbark Weklice, Late Antiquity Lithuania, Wielbark Kowalewko, Bronze Age Latvia, Iron Age Czechia, Bronze Age Estonia, modern day British, modern day Estonia, modern day Iceland and Iron Age Sweden. This result supported the high affinity between the Masłomęcz Group and other Wielbark, Scandinavian and Baltic populations.

**Fig. 3:**
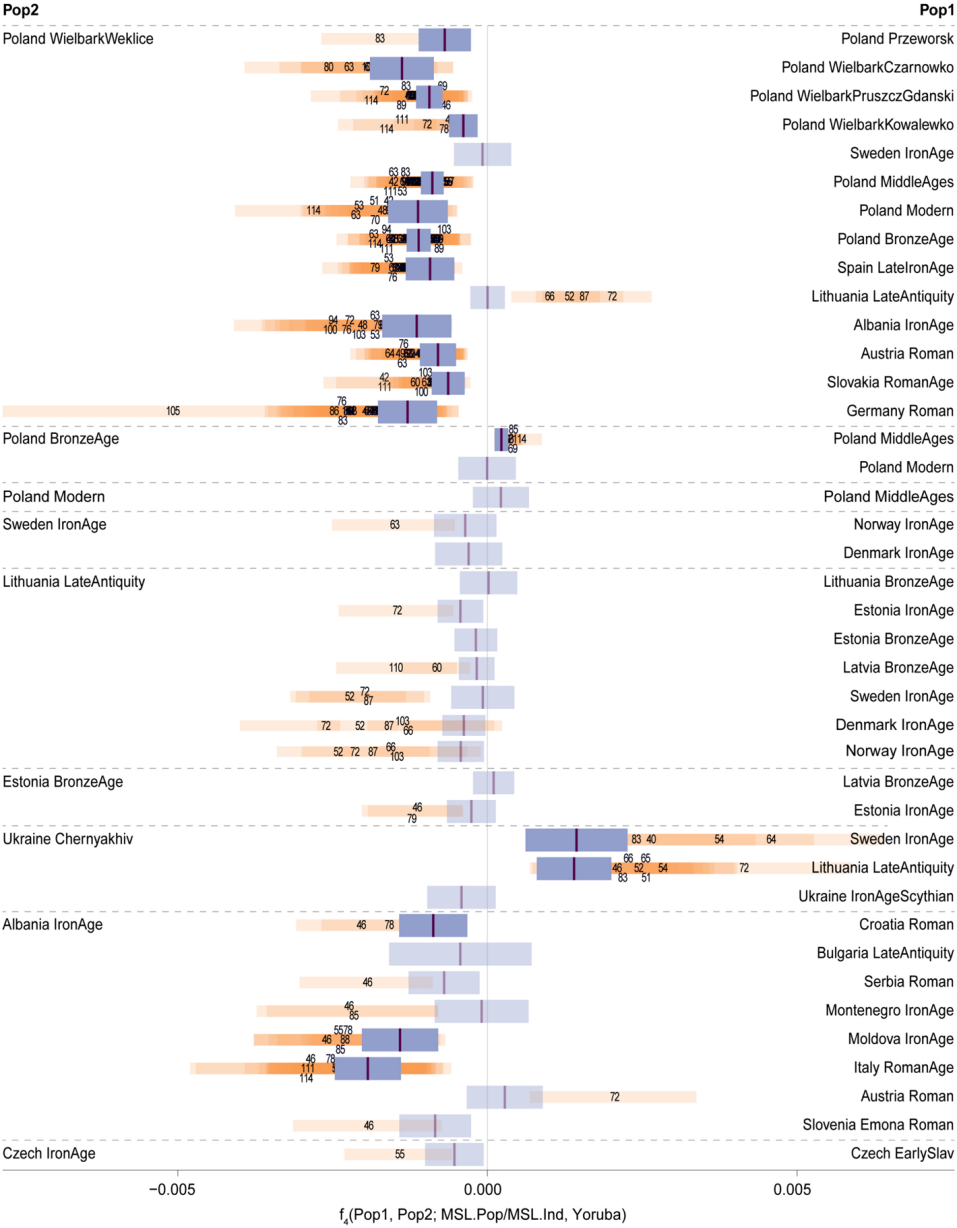
Two population *f*_4_ statistics for the MSL population and individuals. *F*_4_(Pop1, Pop2; MSL.Pop/MSL.Ind, Yoruba) statistics for selected pairs of test populations (full results in Additional file 2: Table S16). Purple boxes show the statistics estimate with 95% confidence interval (±2 standard errors) for the MSL population as a whole with the estimates with |Z-score|< 3 shown in light purple. Additionally, for MSL individuals all the estimates with |Z-score|> 3 the statistics are displayed as orange boxes with the individual ID’s marked at the point jackknife estimate value – at the centre of the respective box (shortened to the last two/three digits for improved readability). Despite the very limited readability of the individual IDs in some cases, we decided to keep them in the figure to underline the differences in number of individuals significantly closer to either of the test populations among the comparisons

Among the published Wielbark populations, the MSL population, as well as a large portion of MSL individuals, were genetically closer to Weklice than to Kowalewko, Pruszcz Gdański, Czarnówko or Przeworsk (Fig. 3), or to Bronze Age, Medieval or modern Poland, or Iron Age populations to the south or the west. However, no support was found for a higher similarity of the MSL population to Weklice against Iron Age Sweden or Late Antiquity Lithuania showcasing the limited resolution in differentiating among these genomic components. The MSL population was found to be marginally significantly closer to the Medieval than to the Bronze Age population from Poland, but no such clear distinction was found between the affinities towards Bronze Age vs Modern and towards Medieval vs Modern Polish population. The affinities of the MSL population could not be discerned among Iron Age and Bronze Age Baltic and Scandinavian populations. Despite the archaeological affinities between the Masłomęcz and Chernyakhiv complexes, MSL population was significantly closer to Iron Age Sweden and Late Antiquity Lithuania than to the published Chernyakhiv group from Ukraine. Among the populations located to the south and west of the Wielbark range the MSL population was genetically closer to Iron Age Albania than to Roman Age Croatia, Roman Age Italy or to Iron Age Moldova but no strong support was found for stronger affinities among other southern or south-westerly populations. Stronger affinity towards either Iron Age or Early Slavic Czechia could also not be rejected, which, again, shows the limits of the resolution of *f* statistics to distinguish ancestry sources within post-Bronze Age Europe.

Next to the genetic affinities of the MSL population as a whole, certain MSL individuals displayed affinities deviating from the population-wide ones (Fig. 3). Individuals PL072, PL087, PL052 and PL066 were significantly closer to Late Antiquity Lithuania than to Weklice, with their affinity towards the former confirmed when compared to the Scandinavian populations. The individuals that in the PCA plot were placed towards the southerly populations – PL085, PL069, PCA114, show stronger affinity with the Medieval than with the Bronze Age population of Poland than did the MSL population as a whole, yet their affinities toward southwesterly populations could not be supported in any of the investigated combinations of test population pairs.

**Fig. 4:**
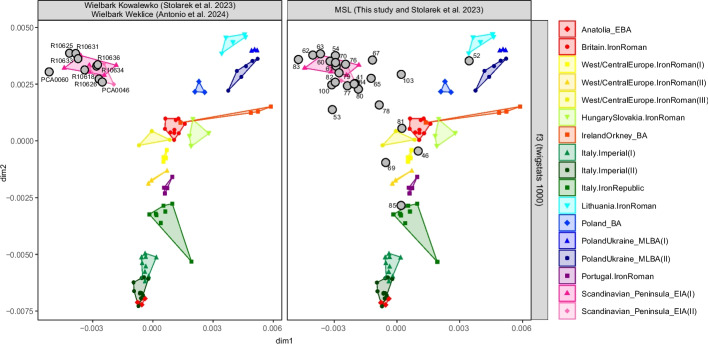
MDS plot of the outgroup *f*_3_-statistics estimated using Twigstats. Each point represents an individual from either previously published Wielbark individuals from Weklice [21] and Kowalewko [18] (left panel, grey markers) or from MSL (right panel, grey markers) or reference ancient individuals from selected populations (colour coded with legend to the right of the panels; range of each group in the plot shaded with respective colour). Only individuals with minimum 0.5× mean genome coverage (shotgun sequencing data only) were included. Modern SGDP Han population was used as outgroup. Individual ID’s for MSL individuals were shortened to the last two/three digits for improved readability

To increase resolution of the *f*-statistics analysis, we applied Twigstats [20] to 25 MSL individuals with minimum 0.5× average genome coverage from shotgun generated (non-enrichment) data. We selected potential source populations following the groupings obtained in the original Twigstats publication (Additional file 2: Table S17). A Multidimensional Scaling analysis for the Twigstats-estimated outgroup *f*_3_-statistics revealed high heterogeneity in the ancestry composition of the MSL individuals, exceeding the heterogeneity among published Wielbark individuals eligible for this analysis (Fig. 4), for which Twigstats was already applied in Speidel et al. [20]. Most MSL individuals grouped with the peninsular Scandinavian groups, but with much broader variation than among the published Wielbark individuals with a distribution gradient towards other populations. Individual PL085 clustered with Iron Age/Republic Italian samples. PL069, PL046 and PL081 grouped with Iron Age and Roman Period West/Central European samples. PL052 grouped with Lithuanian Iron Age and Roman Period samples. A number of individuals were placed intermediate between the Scandinavian and the remaining clusters.

**Fig. 5:**
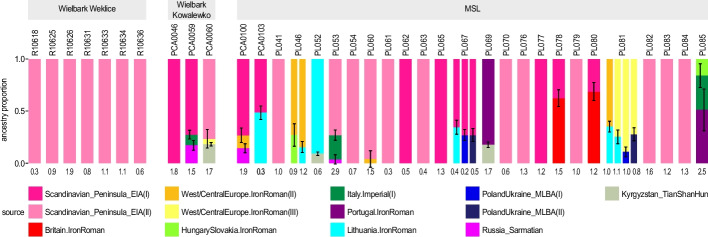
Individual qpAdm analysis for Twigstats estimated *f*-statistics. The estimations were performed for the individuals with minimum 0.5× mean genome coverage (shotgun sequencing data only) from Wielbark Weklice [21], Wielbark Kowalewko [18] and MSL populations. The numbers below the bars present the -log_10_ p-values. For individuals for which *p*-values for multiple models exceeded 0.05 all these models were shown

Individual Twigstat-qpAdm analyses (Fig. 5, Additional file 2: Table S18) again showed that, while most Wielbark individuals (including most MSL individuals) can be modelled using exclusively Early Iron Age peninsular Scandinavia as ancestry source, there are also individuals that require additional sources of ancestry. Thirteen of the 25 MSL individuals included in the analysis, as well as all seven Weklice individuals and one Kowalewko individual, could be modelled as single Scandinavian source. Four MSL individuals showed primary ancestry in Scandinavia but required another source or sources for their ancestry modelling: Iron Age Central European and Sarmatian (PCA0100), Imperial Italian with Sarmatian (PL053), Roman Age Western-Central European (PL060) and Iron Age Lithuanian or Middle-Late Bronze Age Ukrainian (PL067). Two remaining analysed Kowalewko individuals shared this pattern, being modelled as majority Iron Age Scandinavian ancestry plus Roman Italian and Sarmatian (PCA0059) or plus Hunnic Central Asian and Western-Central European (PCA0060) ancestry source. The remaining eight analysed MSL individuals did not seem to have a majority of Iron Age Scandinavian ancestry. Individual PCA0103 was modelled as approximately half Iron Age Scandinavian and half Iron Age Lithuanian ancestry. Two individuals, PL078 and PL080, were modelled as a mixture of a majority of Iron Age British and minority of Iron Age Scandinavian ancestry. A further five individuals showed no signs of Iron Age Scandinavian ancestry at all. Two individuals, PL046 and PL081, appeared to be predominantly of Western-Central European Iron Age/Roman ancestry, although this required a second ancestry source from either Iron Age Hungary, Iron Age Lithuania or Middle-Late Bronze Age Poland/Ukraine. Individual PL052 was best modelled with Lithuanian Iron Age with small addition of Central Asian ancestry, PL069 as Iron Age Portuguese with, again, small addition of Central Asian ancestry, and PL085 as Iron Age Portuguese with Imperial Italian and Iron Age Hungary-Slovakian ancestry.

**Fig. 6:**
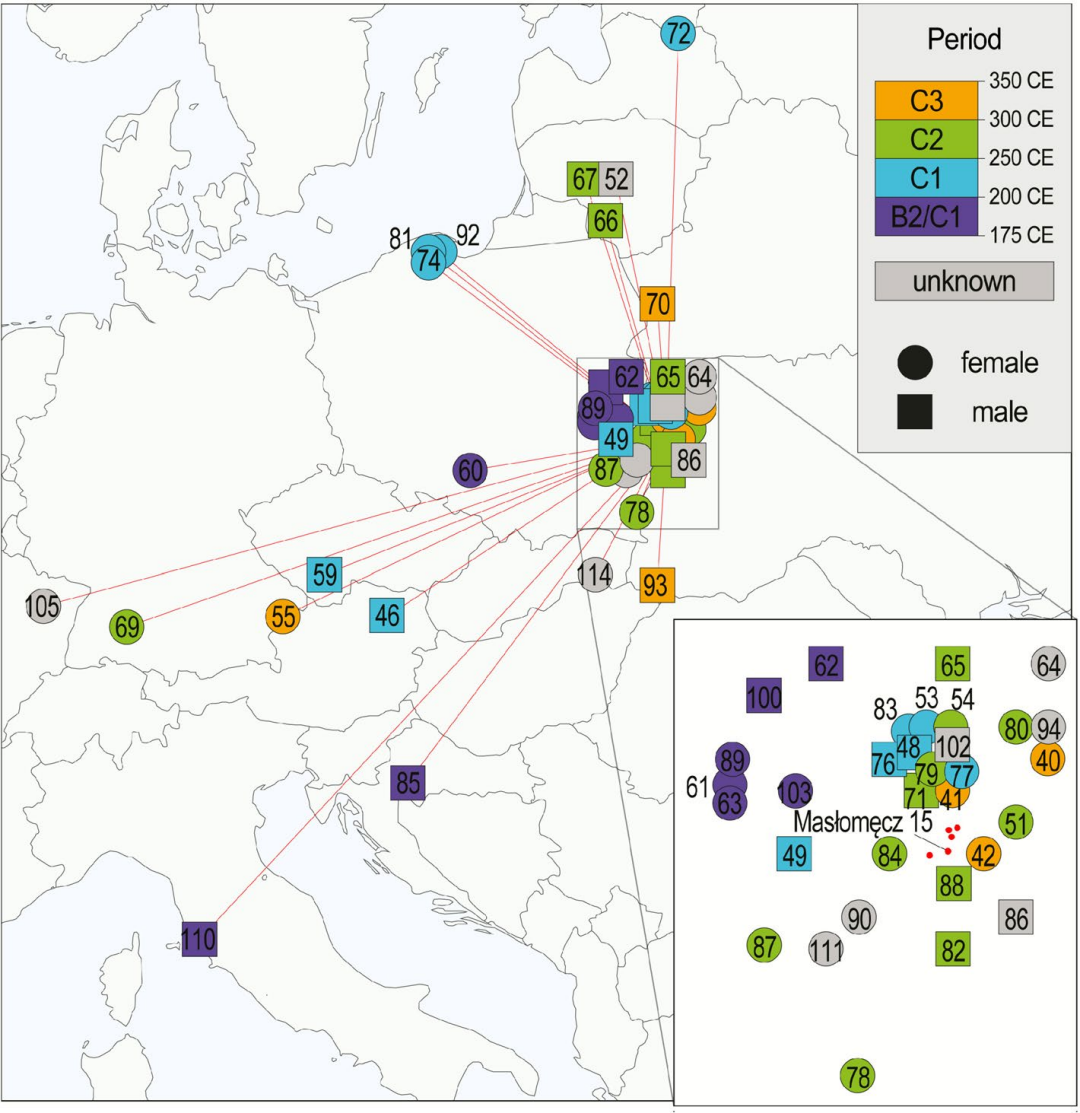
Ancestry origin vectors estimated by mobest. Markers indicate geographic point of the highest probability of occurrence of a given individual’s genomic ancestry around the time of the individual’s life. The high point density area, showing individuals whose ancestry’s highest probability points have been estimated as local was zoomed in in the bottom right. Circle markers denote females and square – males. Marker colours denote the individual’s assignment to an archaeological period (individuals with imprecise dating encompassing more than one period category were coloured with the midpoint period category). Individual labels were shortened for visibility to the two or three terminal digits. Archaeological sites from which all the MSL samples originate are marked with red dots in the zoomed panel (with Masłomęcz 15 – the largest site – labelled)

Spatiotemporal interpolation of the individuals’ ancestry using Mobest, which shows the highest probability locality of the tested genome considering the spatial distribution of genomes in the dataset, coevally to the tested individual, revealed that the ancestry of the MSL individuals fell into essentially four groups. The ancestry of the majority of them has the maximum location probability locally (where MSL individuals were included in learning the field, so “local” here means mostly similar to the MSL population). Three individuals (PL074, PL081 and PCA0092) showed ancestry more characteristic of Pomerania, four individuals (PL052, PL066, PL067, and PL072) of the Baltic states, five individuals (PL046, PL055, PL059, PL069 and PCA0105) of Western-Central Europe and two individuals (PL085 and PCA0110) of the Mediterranean (respectively, Balkans and Italy). Four individuals displayed shifts towards either of these directions but their ancestry’s probability maximum was placed less geographically distantly from the Hrubieszów Basin (PL070 towards Baltic states, PL060 towards Western-Central Europe and PCA0093 and PCA0114 towards the South). Ancestry probability density maps (Additional file 1: Fig. S16) varied visibly between the individuals and generally reflect what is summarized by the ancestry vectors in Fig. 6. Both local and non-local ancestry individuals came from across the Masłomęcz group archaeological phases.

## Discussion

In this study, we employed the state-of-the-art ancient genomic methods to investigate the genetic structure and affinities of a geographically limited, but archaeologically very distinct cultural group. This study represents an initial genetic exploration of the Masłomęcz Group, as skeletal material from another few hundred of individuals are still to be analysed. Despite the very fragmentary sampling of this rich assemblage, we were able to reveal some of the genetic characteristics that in the context of the archaeological and historical knowledge shed more light on these people.

Investigation into the origin of the Masłomęcz Group and other peoples around Central Europe is hindered by the widespread custom of cremating the dead in this region during most of the Iron Age. As ancient DNA is extremely unlikely to survive the conditions within a pyre, our knowledge of any individual’s biological ancestry, as derived from genetic data, is limited to the individuals who have not been cremated. This situation poses a question about whether the individuals available to genetic analysis can be assumed to be representative of the community as a whole. In the Masłomęcz Group the share of inhumations versus cremations varies between cemeteries but the inhumations generally make up the majority of burials and are overrepresented by juveniles [37]. Due to the large proportion of child burials and considering no strong sex bias of our studied cohort, we nevertheless believe that our results reflect the true genetic variation of the Masłomęcz Group.

DNA preservation level among the sampled skeletal material was extremely high with 86% of the sampled individuals yielding sufficient genomic data for population analyses. Although it is certainly not representative of the whole Masłomęcz Group anthropological collection given that the samples were selected based on the bone preservation state, it is highly encouraging for the perspective of pursuing further genomic studies for this fascinating complex.

The high level of mitochondrial variability suggests a rather high influx of females and reflects large population size. Due to the limited resolution of mitochondrial genomic data, it is, however, not possible to assess what proportions of this variability come from the presumed founding Goth group related to Scandinavia, the assimilated local groups and the external contacts. In turn, Y chromosomal diversity suggests that the Masłomęcz Group in its majority comprised of male lineages of typically-Scandinavian origin, but also included a non-negligible proportion of lineages that may reflect populations encountered and assimilated along the migration route from Pomerania, or external migrants. This result confirms, on the one hand, the archaeological and linguistic consensus of Scandinavia as the place of origin of the Goths in continental Europe, although more precise pinpointing of the source population location was not possible at the current state of knowledge. On the other hand, it points to the inclusive nature of this group throughout their expansion. Moreover, the high diversity among the present male lineages (also among the Scandinavian-like portion) suggests that the founding population was not limited to just one or a few male kin groups but rather involved a wider group of largely non-related males.

Considering that the genotyped individuals constitute a very small fraction of the overall individuals buried at the studied cemeteries and the long time of their use (particularly Masłomęcz 15 and Gródek 1C from which most of the studied individuals came), it is not very surprising that few close familial relationships have been detected. The single first-degree relationship was found between the PL076 male and PCA0105 female. Although there were insufficient data to infer the type of this relationship directly from the genetic signal, the mismatch of mitochondrial types excludes mother-son and sibling relations, pointing to PL076 as the father of PCA0105. Interestingly, the male has local ancestry and a Scandinavia-associated Y haplogroup, while the female is a genetic outlier displaying signal of non-local, yet not well-pinpointed, ancestry. The mutual location of these two graves (although the affiliation of PCA0105 sample to a particular burial is uncertain) does not indicate kinship as the key to their positioning within the cemetery.

Interestingly, within grave 20 from Masłomęcz, in which a young female (PL040) was buried with three detached skulls (PL041, PL042 and one unsampled), we detected no first- or second-degree relatedness, with the individuals representing three distinct maternal lineages. Signs of possible third-degree relatedness shared by PL040 and PL042 and by PL041 and PL042 were detected by READv2, but genomic data were insufficient for PL042 to pursue the higher resolution exploration of kinship within this grave and no IBD sharing was found between PL040 and PL041. No kinship was detected between two females, PL079 and PL080, who shared a grave, even though the pair was included in the IBD analysis and thus even further degree relatedness can be rejected. There is therefore no indication that common burials were tightly associated to close biological kinship. Male PL062 and female PL063 buried in one grave (the male sat in a casket and the female lying next to the casket, seemingly snuggling into it) were identified as probable 4^th^ degree relatives from diverse maternal lines. Relationships (defined as pairs producing progeny) between close kin were most probably sporadic but not completely absent as indicated by cousin-level relatedness detected between parents in case of two of the studied individuals.

The Masłomęcz group, as a part of the Goth cultural complex, unsurprisingly shows high levels of Scandinavian ancestry. However, it harbours higher genetic diversity than the other Wielbark groups (although Pruszcz Gdański and Kowalewko groups also comprise a few notable genetic outliers; unfortunately, with insufficient data for our in-depth ancestry modelling) and particularly strikingly higher than Weklice. Individuals of full Scandinavian ancestry, who make up about a half of the studied cohort, are accompanied by individuals genetically similar to coeval individuals from as far as the Mediterranean.

The Bronze Age genetic component from the territory of Poland does not seem to play a major role in the genetic make-up of the studied individuals. These results may be partly caused by the lack of appropriate proxy local Bronze Age populations with genomic data, which due to the widespread cremation customs predating Wielbark groups in the regions can probably only partially be addressed in the future. However, it also tentatively points to discontinuity between the earlier inhabitants of the region and the Wielbark and Masłomęcz groups.

Despite the pronounced presence of non-local ancestry in some individuals, they all display close affinity to the MSL population. None of the individuals are significantly genetically closer to other group than it is to MSL as indicated by the *f*_*4*_-statistics (although in some cases the affinity to MSL is not significantly greater than the affinity to another population). This suggests that these individuals, including the genetic outliers such as PCA0110, PCA0105, PL085 or PL069, are nonetheless not simply newcomers from the outside but probably rather represent already consecutive generations of offspring between the local and non-local individuals.

The exact geographical pinpointing of the source of exogenous ancestries detected in some of the individuals is in some cases difficult due to the limitations of the comparative data used as proxy for the actual source populations. We thus caution the reader not to interpret the labels of the used population proxies as literal sources of ancestry of the studied individuals. Nevertheless, the revealed Balkan components seem to align with the proposed presence of Dacians among the Masłomęcz Group and the Central Asian signal can be attributed to Sarmatian connections. The most represented “foreign” genetic component is, however, associated with Baltic ancestry, which, again, very well corroborates archaeological evidence.

The temporal pattern of the ancestry composition of the Masłomęcz Group indicates that contacts with distant populations were characteristic for the Masłomęcz group from its formation rather than slowly expanding through time. Individual PL085, for example, who represents one of the most pronounced genetic outliers towards south-European groups, is dated to period B2/C1, i.e., at the very beginnings of the Masłomęcz group record. Although there was no burial inventory that could suggest nomadic cultural affiliation, the outstanding genetic ancestry drew attention to this individual’s unusual burial position, with legs crossed. Interestingly, Ukrainian genomic data published after the analyses for this study [38], showed that some individuals associated with the Chernyakhiv culture show a genomic signal (at least as observable at a PCA level) very similar to that of the prominent PL085 outlier and these were modelled in qpAdm as 100% Early Iron Age Thracian Hallstatt ancestry, which gives a suggestive indication for this individual’s population affinities.

Individual PCA0110 – the most prominent genetic outlier – was also dated to rather early stages of Goths’ presence in the Hrubieszów Basin. These results point to the cosmopolitan character of the Masłomęcz Group from its early formation. The southerly ancestry influx in the B2/C1 period could be linked to the possible involvement of Goths in the Marcomannic Wars and the associated contact with Sarmatians, as inferred from archaeological and historical evidence [39]. In the C1 period we observe on one hand higher affinities to Scandinavia which could indicate some additional gene flow from the North as well as a signal of south-westerly links with ancestries observed towards Czechia and Austria, in concordance with archaeological evidence of the Gothic presence in the Bohemian Basin in this period [40]. The appearance of Balt-associated artefacts in grave inventories in the C2 period is accompanied by individuals with well-detectable Baltic ancestry [7].

Although we detected an effect of sequencing strategy (shotgun vs target enrichment) causing clustering of samples in some of the allele-frequency-based analyses, our tests suggest that this bias generally did not affect the estimates of affinities towards external groups. We thus believe that the results of our population genomic inferences are largely unaffected by the enrichment bias.

## Conclusions

The striking genomic variation evidenced by this study gives us unprecedented confirmation that the complexity on the biological level mirrors well the astounding cultural complexity of the Masłomęcz Group. The ties between the Goth and Baltic groups, which have long been observed in archaeological evidence, has now been corroborated by the presence of individuals displaying partially Baltic ancestry among the persons buried within the Masłomęcz Group community. Individuals characterized by more “steppe” ancestries corroborate the presence of Sarmatians among the Masłomęcz Groups as known from archaeological data. Moreover, the burial from Strzyżów, which included an artefact as only found in the Balkans, indeed exhibited distinct Balkan-related ancestry [41].

Our study also seeks to address some of the issues raised along with previous publications of genetic and genomic data from the Masłomęcz Group [17, 18] as the archaeological affiliation of some the material analysed in these studies has been contested [6] and the interpretation of the biological affinities of this group with other populations challenged [19, 20]. Contrary to these previous studies, we do not observe much genetic continuity in the region of present-day Poland although precise modelling of the level and directions of gene flow processes that were shaping the genomic structure in the Oder and Vistula basins will require further research. Nevertheless, the example of Masłomęcz Group paints a picture of a very dynamic landscape of high levels of long-range mobility and with notable turnovers of the core genomic ancestries, albeit often biologically challenging to distinguish.

Our results corroborate the hypothesis based on archaeological data that the Masłomęcz Group was both culturally and genetically highly complex. It built upon the mixture of Northern ancestry brought by the Goths migrating South-East and the local components of the people they encountered on the way, but was also highly permeable to external individuals. This high variation characterized the group already at its formation in the late 2^nd^ century CE and was sustained throughout their presence in the Hrubieszów Basin until the mid-4^th^ century CE. Although the non-Scandinavian genetic ancestry components already appear at the earliest stages of Goth continental expansion (probably including both local assimilations and geographically-distant contacts), this genomic variation appears to peak with the Masłomęcz Group as compared to other Wielbark populations. The identified directions of extensive contacts of the group point, among others, to the Balkans and Baltics but possibly also Western-Central Europe. With the constantly growing amount of comparative ancient genomic data, future studies should be able to increase the precision of source identification for these contacts.

We found no clear relation between the genetic relatedness among the individuals and either burial in a common grave nor the grave location. Understanding of the complex burial customs of the Masłomęcz Group would require denser sampling throughout the cemeteries and is a promising prospect for future studies.

Our analyses additionally corroborate that at the studied period (2^nd^ – 4^th^ century CE) the resolution to discern particular ancestry sources, especially among Scandinavia, Poland and the Baltics, is quite limited. Further data (particularly such of high coverage) from these regions and period are required to trace the directions and intensities of gene flow with more accuracy and precision; in particular, to pinpoint the origin of the Scandinavian ancestry detected in MSL and other Wielbark populations to a particular region within Scandinavia. Additionally, crucial determination of the proportion of the freshly incoming Scandinavian versus the local Oksywie ancestries in the formation of the Goth groups might never be resolved due to the scarcity of skeletal burials. Nevertheless, contrary to archaeological interpretations, which postulated Central Pomerania (zone C of the Gothic settlement and proposed location of Gothiscandza, [5]) as the continental place of origin of the Goth migration towards Hrubieszów Basin, our results rather point to the Elbląg Upland (zone D) with Weklice site displaying the highest affinity to the Masłomęcz Group among the published Wielbark sites, as the best proxy source population.

The transdisciplinary approach presented here has opened an opportunity to test the hypothesis based on the archaeological evidence describing the Hrubieszów Basin phenomenon as open and culturally tolerant. The inhabitants of this region not only adapted elements of foreign material and spiritual cultures but also physical representatives of other cultures. The identification of the origin of these “foreigners” opens up a completely new page of the history of barbarian Europe, challenging the common understanding that relations between particular peoples were predominantly antagonistic. On the other hand, the remarkable achievement of the genetic approach is demonstration of a rapid adaptation of the “aliens” to local cultural norms.

## Methods

### Laboratory processing of the samples

Skeletal material from Masłomęcz Group was sampled as a part of a wider project and thus the research presented here was not designed as a comprehensive analysis of this archaeological assemblage, but rather as an initial stage of investigating its genomic landscape. Samples from 43 individuals from Masłomęcz Group (Additional file 2: Table S1) were collected at the Maria Curie-Skłodowska University in Lublin and transferred to the ancient DNA facilities of the Laboratory of Paleogenetics and Conservation Genetics, Centre of New Technologies, University of Warsaw. For 41 individuals at least one petrous part of temporal bone was sampled; for eight of these, a tooth sample was additionally collected. For two individuals (PL042 and PL070), only tooth samples were available and sampled for DNA analyses. In the clean room facility each bone sample was cleaned of surface debris with a dry sterile toothbrush, UV-irradiated for 15 min on each side and cut in half through the centre of the petrous part with a cutting disc. Between 20 and 100 mg of bone powder was collected by drilling in the densest part as indicated by Pinhasi et al. [42]. Each tooth sample was washed with ca. 2% sodium hypochlorite, then twice with milliQ water and UV-irradiated for 15 min on each side. The surface of the tooth was cleaned off with abrasive diamond wheel point and the cementum powder (30–65 mg) was sampled by diamond drill bits sanding.

DNA was extracted using the Rohland et al. [43] protocol with the final elution 2 × 25ul resulting in 50ul of DNA extract. Double-stranded DNA libraries were prepared using a modified Meyer and Kircher [44] protocol with varying UDG treatments (see Additional file 2: Table S2). For chosen samples additionally single-stranded libraries were prepared according to Gansauge et al. [45]. For each individual between 3 and 11 dual-index libraries (with no shared indices between index pairs within each sequencing run) were prepared. The libraries were first shallow-sequenced using NextSeq550 and v2.5 150 cycles kit (Illumina) with ca. 1M reads targeted to assess endogenous DNA level and deamination-derived damage to the molecule ends. Libraries exhibiting minimum of 1% endogenous DNA level were further deep-sequenced with NovaSeq6000 and the S1 2 × 50 cycles kit (Illumina) using either shotgun approach or following an enrichment protocol. Selected libraries were enriched using the Arbor Biosciences Human Affinities Prime Plus panel, which includes i.a. the 1240k SNP set [46], according to the manufacturer protocol. Selected male libraries were additionally enriched using a panel of ca. 10 000 Y-chromosome-lineage informative SNPs, in-house designed [47] and custom-made by Arbor Biosciences.

### Sequencing reads processing and authentication

Sequence reads were curated and analysed using an in-house pipeline as follows. Adapter sequences were removed and paired-ends collapsed using AdapterRemoval2 [48]. Fastq files with removed adapters, with no filtering, for all libraries were uploaded to the European Nucleotide Archive (ENA) under project number PRJEB74770 [49]. The reads were then mapped to hs37d5 human reference genome using bwa mem [50], filtered for minimum mapping quality of 30 using samtools view [51] and duplicate reads were removed using picard MarkDuplicates [52]. Mapping statistics were obtained using samtools idxstats, samtools flagstat and Qualimap [53]. The libraries were merged across each individual after clipping 5 nt off both ends for the non-UDG treated libraries and 2 nt for the half-UDG-treated libraries using BamUtil’s trimBam function [54]. The final merged bam files for each individual were also uploaded to the ENA PRJEB74770 project [49].

Level of the deamination-derived damage on the terminal nucleotides of DNA molecules was estimated for each pre-merging library using mapDamage2 [55]. Contamination level was assessed for each merged library, as well as for all pre-merging libraries for which it was possible, using a number of approaches. Schmutzi [56] and ContamMix [57] were used for mitogenome-mapped reads. ANGSD [58] was used for male individuals to estimate the observed level of X-chromosome heterozygosity. HapCon [59] and hapCon_ROH [60] were used to estimate, respectively, X-chromosome and autosomal, contamination level based on the analysis of the detected runs of homozygosity.

Genetic sex was determined using shotgun data only basing on relative coverage between sex chromosomes and autosomes [61] as well as on the proportions between reads mapping to either X or Y chromosome [62].

Mitochondrial genome sequence for each individual was reconstructed using separate mapping using both rCRS (GenBank Acc. NC_012920) and RSRS (inferred ancestral mitochondrial genome to all living humans) [63] human mitochondrial reference genomes following the same approach as in case of the whole-genome mapping described above. Consensus sequence was called for sites with minimum coverage by three reads using bcftools functions mpileup and consensus [51] as well as bedtools functions genomecov and maskfasta [64]. Haplogroup was determined using Haplogrep2 [65] both with rCRS and RSRS as reference. Only haplogroup assignments concordant between the two references were accepted.

Y chromosome haplotypes were determined using Yleaf software [66] with the following parameters: -r 1, -q 20, -b 90. The analysis was based on the 73 487 SNP panel from the International Society of Genetic Genealogy (isogg.org) as well as on a 263 555 SNP panel from the Yfull.com database. Data from the publicly available collections at www.yfull.com and www.familytreedna.com were used to determine the contemporary distribution of Y chromosome haplogroups. Final haplogroup assignments were manually curated to ensure accuracy.

Merged bam files of the positively authenticated individuals were genotyped for the 1240k SNP panel. Pileup file was generated using samtools mpileup (-R -B -aa -q30 -Q30) and pseudohaploid genotypes were called using sequenceTools pileupCaller (https://github.com/stschiff/sequenceTools) by choosing random allele for each target SNPs covered by at least one read. A threshold of 10k genotyped SNPs was applied for including individuals in further analyses. The pseudohaploid genotype dataset was uploaded to the Poseidon public repository [67].

### Dataset assembly

For comparative analyses we assembled a dataset of 1240k data for 2422 ancient and modern individuals from relevant populations, including Yoruba population to be used as outgroup, for which genomic data (at least 10 000 SNPs genotyped from the 1240k panel) was available as a part of AADR v54.1 dataset [68]. Additionally, we retrieved data for 356 ancient individuals from Poland not included in this version of AADR set [18, 69] from the European Nucleotide Archive (PRJEB53670, PRJEB48333) as fastq raw files and we processed them using the same bioinformatic pipeline as the newly obtained sequencing data in this study. We merged the pseudohaploid genotypes using Eigensoft [70]. The assembled dataset (including the 37 newly sequenced individuals; hereafter, “1240k dataset”) comprised in total 2815 individuals grouped into 89 “populations” (Masłomęcz Group individuals, Yoruba, and 87 relevant reference populations; Additional file 2: Table S9). Additionally, 522 modern West Eurasian individuals from Lazaridis et al. [35] were merged with the “1240k dataset” and this merged dataset (thereafter “HO dataset”) was subsampled to Human Origin SNP set (621 799 SNP positions) for which the modern individuals have almost complete genotype data. The “HO dataset” (3337 individuals total) was used in the analyses sensitive to data missingness (see further).

Individuals from the Masłomęcz 15 archaeological site for which genomic data (at least 20 000 1240k panel SNPs genotyped) was published in Stolarek et al. [18] (labelled PCA0088 – PCA0114; Additional file 2: Table S5) were analysed together with the newly sequenced individuals. One sample – PCA0113 – was identified as a duplicate of the PL085 sample by the applied kinship analyses (Additional file 2: Table S7). This finding was further corroborated with shared both mitochondrial (V) and Y chromosome (J2b) haplogroups. Inspection of the information available for the samples revealed that the two samples come from the same grave (Masłomęcz 15, grave 260). PL085 was sampled from petrous bone sampled from a complete braincase, PCA0113 from a tooth. PCA0113 was thus excluded from all further analyses. The final joint Masłomęcz group dataset, hereafter “MSL”, in total comprised 50 individuals (37 newly sequenced and 13 individuals with from Stolarek et al. [18]).

### Kinship analysis

Familial relationships between the individuals were inferred using READv2 [30], BREADR [31], NGSrelate [32] and ancIBD [33] applied to the full MSL dataset, including the PCA0113 sample that as a result of these analyses turned out to be a duplicate of PL085.

To estimate relatedness based on the analysis of the Identity By Descent (IBD) tracts we subjected each individual’s human-reference-mapped reads to a custom-built pipeline. First, we recalibrated base quality across the untrimmed reads based on the post-mortem damage patterns using ATLAS [71]. Then, we performed phasing and imputation using GLIMPSE [72, 73]. The imputed dataset comprised 1 086 711 SNPs from the 1240k panel across chromosomes 1 to 22. Only individuals that surpassed the threshold of minimum 500 000 genotyped SNPs were included in the further IBD calling using ancIBD [33].

The level of consanguinity among the studied individuals’ parents was investigated using ROH segment length distribution estimated using hapCon_ROH [59].

### Population genomics

Principal component analysis was carried out for the “HO dataset” (Additional file 2: Table S9) using modern West Eurasian individuals from Lazaridis et al. [35] with minimal data missingness for PC estimation and ancient individuals (as well as one modern individual from Poland) were projected onto the estimated space. We used Eigensoft smartpca (newshrink: YES, lsqproject: YES, outliermode: 2).

For the Admixture analysis the “HO dataset” was filtered using PLINK v1.9 [74] for minimum minor allele frequency (–maf 0.01) and linkage disequilibrium (–indep-pairwise 200 5 0.5). The analysis was performed using Admixture v1.3 [75] in unsupervised mode for K between 2 and 8, with modern West Eurasian individuals used for clustering and ancient samples later projected onto the estimated component landscape with 200 bootstrap replicates.

All *f*-statistics for the were estimated for the “1240k dataset” (Additional file 2: Table S9) using Poseidon Xerxes [76]. Outgroup *f*_3_ statistics were estimated using “F3vanilla” function in Xerxes in form: *f*_3_(Test.Pop1, Test.Pop2; Yoruba) between all populations in the dataset, as well as with the MSL population split into single individuals. These were transformed into dissimilarity matrix (1- *f*_3_), subjected to multidimensional scaling (MDS) using R package vegan::wcmdscale [77] and plotted using R. A neighbour-joining tree using 1-*f*_3_ values was estimated using ape::nj R package [78] and plotted using R basic [79] in form of a cladogram (as the estimated branch lengths resulted in a highly illegible tree with all nodes squeezed tightly near the root and long terminal branches due to minimal variation in the outgroup *f*_3_ statistics values in the dataset). Bootstrap support values were obtained using the approach from Atağ et al. [80] by dividing the genotype data to chunks of 30,000 SNPs, randomly sampling chunks with replacement and recalculating *f*_3_ statistics. Bootstrap support values for the original dataset tree topology were calculated using TreeAnnotator from the Beast 2.6.6 package [81].

Admixture *f*_3_ statistics were estimated using “F3vanilla” function in Xerxes in form: *f*_3_(Test.Pop1, Test.Pop2; MSL.Pop) for MSL as a group as well as *f*_3_(Test.Pop1, Test.Pop2; MSL.Ind) for each MSL individual.

*F*_4_ statistics were estimated in a variety of forms to investigate the presence of outliers, i.e. “outlier *f*_4_” – *f*_4_(MSL.Pop, Test.Pop; MSL.Ind, Yoruba); the homogeneity of the MSL population, i.e. “homogeneity *f*_4_” – *f*_4_(Yoruba; Test.Pop; MSL.Ind1, MSL.Ind2); the homogeneity of other Wielbark populations, i.e. “homogeneity *f*_4_” – *f*_4_(Yoruba; Test.Pop; WielbarkPopulationA.Ind1, WielbarkPopulationA.Ind2); and the relative affinities of the MSL individuals and the MSL population to pairs of test populations, i.e. “two population *f*_4_” – *f*_4_(Test.Pop1, Test.Pop2; MSL.Ind/MSL.Pop, Yoruba).

For Twigstats analyses we merged GLIMPSE imputed ancient genomes of coverage > 0.5× with previously published ancient genomes (Additional file 2: Table S17) and SGDP high-coverage present-day genomes and applied Relate [82, 83] to infer genealogies. We restricted our analysis to transversions and used a 4 × 10^–9^ per base per generation mutation rate. We then used Twigstats [20] to compute *f*-statistics with a time cut-off of 1000 generations (which was previously shown to perform well for datasets comprising similar time period and region [20]). We quantified fine-scale population structure by computing *f*_3_-statistics between each pair of individuals, choosing present-day Han in SGDP as the outgroup. We then ran a Multidimensional Scaling analysis (MDS) on the matrix storing 1-*f*_3_ for every pair of individuals to quantify population structure. We additionally inferred high resolution ancestry models by computing Twigstats *f*_2_-statistics supplied to admixtools2 [84]. We used a rotational qpAdm approach with outgroups Russia_Shamanka_Eneolithic, Anatolia_EBA, IrelandOrkney_BA, Yamnaya and putative source groups Britain.IronRoman, CentralEurope.IronRoman(I), CentralEurope.IronRoman(II), CentralEurope.IronRoman(III), HungarySlovakia.IronRoman, Italy.Imperial(I), Portugal.IronRoman, Lithuania.IronRoman, PolandUkraine_MLBA(I), PolandUkraine_MLBA(II), Scandinavian_Peninsula_EIA(I), Scandinavian_Peninsula_EIA(II), Russia_Sarmatian, Kyrgyzstan_TianShanHun [20].

We used Mobest [85] to investigate the spatiotemporal distribution of the ancestry patterns found in the studied individuals. As input data we used PC1 and PC2 coordinates obtained from smartpca Principal Component Analysis described above. Only individuals located between 30° and 70° latitude and −25° and 70° longitude were retained for the analysis (Additional file 2: Table S19). We used dsx = dsy = 800*1000 and dt = 200 kernel size. We interpolated the distributions coevally with each analysed individual (relative time 0). MSL individuals were not excluded from the field learning process and hence “local” ancestry is predominantly informed by the MSL genetic signal.

### Enrichment bias

The effect of applying target enrichment procedure on the population genomic analyses was observed in the outgroup *f*_3_-statistics-based neighbour-joining tree. The extent of this bias was investigated using additional *F*-statistics analyses: 1) outgroup-*f*_3_(Test.Pop1, Test.Pop2; Yoruba) among all the reference populations and the MSL population split into MSL.Shotgun.Pop and MSL.Enriched.Pop subgroups, to investigate whether the two subgroups relate differently to the reference populations than the full MSL population; 2) *f*_4_(MSL.Shotgun.Pop, MSL.Enriched.Pop; MSL.Shotgun.Ind, Yoruba), *f*_4_(MSL.Enriched.Pop, MSL.Shotgun.Pop; MSL.Enriched.Ind, Yoruba), *f*_4_(MSL.Shotgun.Pop, MSL.Pop; MSL.Shotgun.Ind, Yoruba) and *f*_4_(MSL.Enriched.Pop, MSL.Pop; MSL.Enriched.Ind, Yoruba) to test whether the individuals from each of the two subgroups are significantly closer to their own subgroup than to the other subgroup or to the MSL population as a whole; 3) *f*_4_(Yoruba, MSL.Shotgun.Pop; MSL.Shotgun.Ind1, MSL.Shotgun.Ind2) and *f*_4_(Yoruba, MSL.Enriched.Pop; MSL.Enriched.Ind1, MSL.Enriched.Ind2) to test whether individuals within each of the two subgroups display similar level of affinity to their respective subgroup, i.e. testing the homogeneity of the two subgroups; 4) *f*_4_(MSL.Enriched.Pop, MSL.Shotgun.Pop; Test.Pop, Yoruba) to test whether the two subgroups display different levels of affinity towards reference populations; 5) *f*_4_(MSL.Shotgun.Pop, Test.Pop; MSL.Shotgun.Ind, Yoruba), *f*_4_(MSL.Enriched.Pop, Test.Pop; MSL.Enriched.Ind, Yoruba) to test the presence of outliers within these two subgroups, i.e. individuals for which there exists a reference population closer than their respective subgroup; 5) *f*_4_(MSL.Enriched.Pop, Test.Pop; MSL.Shotgun.Ind, Yoruba), *f*_4_(MSL.Shotgun.Pop, Test.Pop; MSL.Enriched.Ind, Yoruba) to test whether the significant affinity of the individuals towards the MSL population against the reference populations is driven by the enrichment treatment or it persists even when the MSL population only comprises the opposite-treatment individuals.

## Supplementary Information

Additional file 1: Supplementary text with descriptions of the archaeological sites (including references to relevant literature [2, 6, 7, 9, 10, 14, 15, 18, 19, 33, 36, 41, 56–60, 143–151]), details for the used data quality filtering and the description of the investigation of the enrichment bias detected, as well as Supplementary Figures S1-S16.

Additional file 2: Supplementary Tables S1-S19. Containing metadata of the used material and detailed results of applied formal analyses. Please note that due to their large size, Supplementary Tables 13, 14 and 16 are available through Zenodo [152].

## Data Availability

Sequence data that support the findings of this study have been deposited in the European Nucleotide Archive with the primary accession code PRJEB74770 [49] and in the Poseidon Community Archive as “2026_Golubinski_Maslomecz” package [67]. Genotype data used in population genomic analyses has been sourced from [18, 21–23, 26, 35, 46, 69, 86–141] (includes datasets accessed through AADR [86]). Mitochondrial haplogroup frequencies data was sourced from [18, 69, 142].
