# Supplementary material for: Cosmopolitanism in the depths of Barbaricum evidenced by archaeogenomic data from the Late Iron Age Goth community of the Masłomęcz group
Source: Genome Biol. 2026 Jan 28;27:50. doi: 10.1186/s13059-026-03969-4 (PMC12924530; doi:10.1186/s13059-026-03969-4)
Supplement: Supplementary file 1 — Additional file 1: Supplementary text with descriptions of the archaeological sites (including references to relevant literature [2, 6, 7, 9, 10, 14, 15, 18, 19, 33, 36, 41, 56–60, 143–151]), details for the used data quality filtering and the description of the investigation of the enrichment bias detected, as well as Supplementary Figures S1-S16. [file 13059_2026_3969_MOESM1_ESM.pdf]

Supplementary Text and Figures for

# Cosmopolitanism in the depths of Barbaricum evidenced by archaeogenomic data from the Late Iron Age Goth community of the Masłomęcz group.

Michał Golubiński *et al.* 2026, *Genome Biology*

*Corresponding author. Email: martyna.molak@gmail.com

## Supplementary Text 1. Archaeological sites

### Masłomęcz 15

The cemetery occupies the southern slope of a prominent elevation, located about 120-150 m from the large settlement to the east. The site is perfectly preserved with no traces of damage. It was established on a rectangular plan with corners on the EN-WS axis. The central part was occupied by a circular plaza within which traces of the roots of four trees, a round, cased object ("holy well") and a rectangular object - "corpse storage", were found. The graves to the west and east sides of the plaza were arranged on NW-SE axes; to the south of it on arches. In the NE part of the cemetery there was a circle formed by graves with a grave in the middle. In the C3 phase, a part of the graves to the SW of the plaza with trees was levelled, and a circle 13 m in diameter was delineated (probably "bounded" by a fence or low rampart), in the centre of which a light wooden structure was set up. Around the structure and within the circle, burnt human remains were placed, which formed a layer of several centimetres. At the same time, to the south of this ossarium, a layer formed by metal clothing parts and broken vessels.

532 burials (a piece of the cemetery, where there may be about 30 graves, was left unexcavated): 50 cremations, 13 cremation-skeletal, 245 full skeletal, 212 partial skeletal; 34 graves comprising more than one deceased; 224 children; in 18 cases the deceased were accompanied by skeletons of birds or fish (some are burials of animals only); 43 objects are symbolic graves, did not contain bones; in 96 cases the grave was found reopened, sometimes several times. The dead were buried on their backs, on their sides, on their stomachs and seated. Some of the dead were accompanied by skeletal parts of other individuals. The dead lay at the bottom of grave cavities without casing; on organic bedding, in log coffins, in box coffins, on marls/stretchers; laid on boards, deposited in a groove hollowed out in the bottom of the grave marring the coffin. Boat burials are an exception. Children were sometimes buried in baskets. Some graves had a wooden chamber structure, in some only a 'canopy' was found; in one case the walls of the chamber were hung with skins or mats.

Dating: B2b - D1 (last quarter of the 2$^{nd}$ century to the end of the 4$^{th}$ century CE).

GPS coordinates: 50.722 N, 23.893 E

Key literature: [2,7,9,10,143–147]

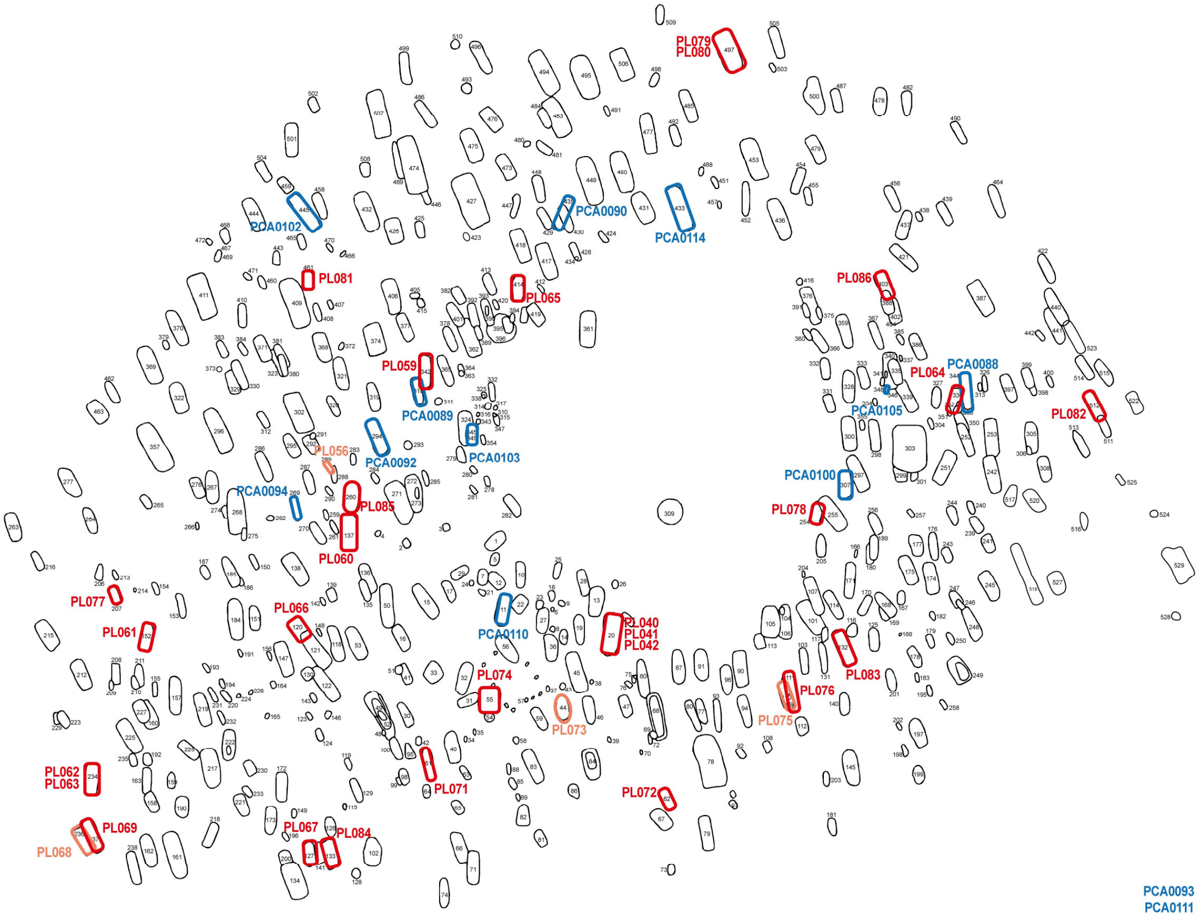


Fig. S1. Map of Masłomęcz 15 site. Individuals included in the study are indicated by red grave contours and labels (orange for the individuals excluded from the genetic analyses due to insufficient data or contamination). Previously published individuals are indicated by blue grave contours and labels. Individuals PCA0093 and PCA0111 could not be reliably traced to any particular grave. Affiliation of some other published individuals, i.a. PCA0103 and PCA105, to their respective graves as indicated in [17] and [18] may not be accurate [6,19]. Figure credit: Bartłomiej Bartecki.

**Gródek 1C**

Cemetery located on top of a flat loess spur lying along the Bug River in its southern part. Only a fragment of the cemetery, estimated to be 1/3 of the area, was excavated. The site in its upper parts was destroyed by intensive agricultural works and fortifications of the defence line from the Polish-Soviet war. Three circles delineated by graves were noted – each with a burial in the centre. In the SW part of the site remnants of a layer of cremated remains was revealed. 192 graves were examined: 12 cremation, 1 cremation-skeletal, 1 animal, among the skeletal burials 68 were children's. Animal skeletons were found in the graves. One burial is of Sarmatian, niche character.

Dating: B2/C1-D1.

GPS coordinates: 50.799 N, 23.949 E

Key literature: [14,148]

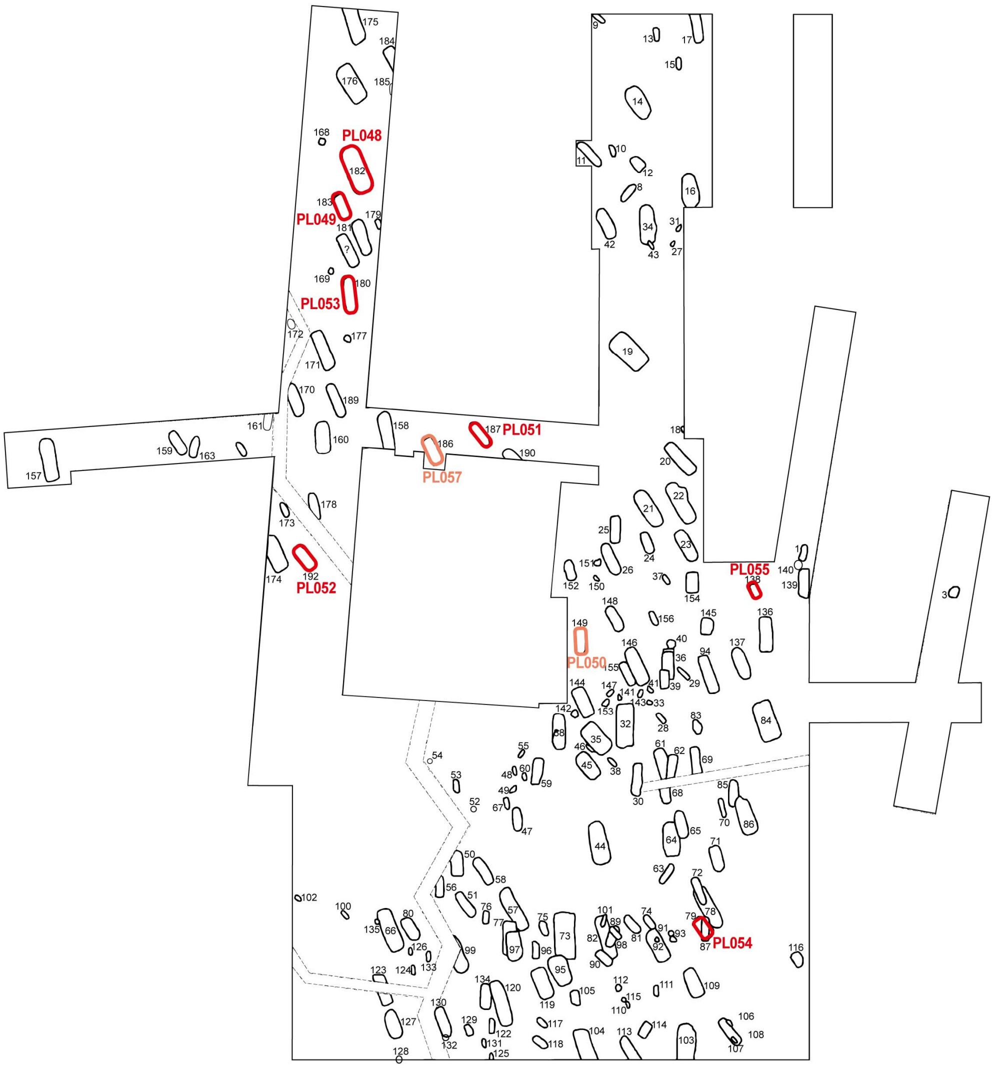


Fig. S2. Map of Gródek 1C site. Individuals included in the study are indicated by red grave contours and labels (orange for the individuals excluded from the genetic analyses due to insufficient data or contamination). Figure credit: Bartłomiej Bartecki.

**Moroczyn 25**

A small cemetery established on a small clear elevation, the top of which was marked by a “totem pole”. The central part destroyed by a modern dig. Graves: 5 cremation burials without urns, 3 skeletal (including 1 child), 3 symbolic - without bones. In one of the graves animals were deposited with the deceased. Dating: single grave C2b? – second half of 3rd cent CE; remaining graves C3-C3/D1 – 300-350 CE. GPS coordinates: 50.840 N, 23.937 E. Key literature: [15,149].

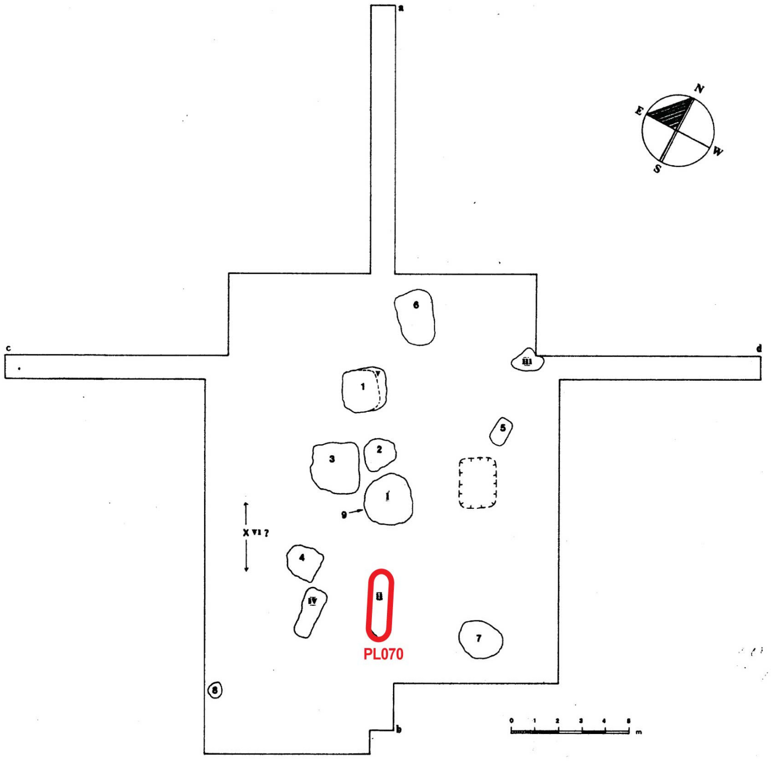


Fig. S3. Map of Moroczyn 25 site. I – V skeletal graves, 1 – 9 cremation and “symbolic” graves. Figure credit: Andrzej Kokowski.

**Strzyżów**

A single grave (sample ID: PL046), rather outside the cemetery, skeletal. Distinguished by the presence of a gilded silver cosmetic spoon with an analogue in Hungary. Dating: C2. GPS coordinates: 50.843 N, 24.014 E. Key literature: [41,150].

**Werbkowice Kotorów II**

A single grave (sample ID: PL087) in a wooden chamber, established outside the "proper" cemetery. Dating: C2b – ca. 300 CE. GPS coordinates: 50.719 N, 23.724 E. Key literature: [151].

## Supplementary Text 2. Data quality filtering

Analysis of damage patterns in the obtained libraries confirmed (at least predominantly) ancient provenance of the extracted human DNA with terminal base deamination rate between 0.5 and 24% depending on the UDG treatment applied (Additional file 2: Table S2, Fig. S7). Due to low number of sequencing reads obtained contamination analyses were not possible to perform with either of the applied methods for four samples (PL050, PL056, PL068, and PL073; Additional file 2: Table S3). From the remaining 39 samples, in four the contamination estimate exceeded 5% in an estimate by at least one method. Sample PL042 was estimated to comprise between 4.2 and 7.4% nuclear contamination using hapCon_ROH [60], but because this estimate did not exceed 10% and the sample was not found contaminated on mitochondrial level by neither Schmutzi [56] nor ContamMix [57], it was not excluded from further analyses. For sample PL048, the library with high contribution to the final bam Schmutzi analysis resulted in between 6 and 10% contamination estimate, however, ContamMix, ANGSD [58] and hapCon [59] did not find contamination above 2% and so we retained this sample in the final dataset. ContamMix estimated mitochondrial contamination for sample PL051 between 4.7 and 8.4%, but considering that maximum estimate using hapCon_ROH (in a single 12cM ROH) did not exceed 1.5%, we decided to retain this sample in the final dataset. For PL057 ContamMix, ANGSD and hapCon estimates exceeded 5% (at between 8.7 and 14.3%, 4.1 and 6.7%, and 4.4 and 6.3%, respectively) and thus this sample was excluded from any further analyses.

## Supplementary Text 3. Enrichment bias

$F_3$ statistics neighbour-joining tree (Fig. 2C) revealed, quite strikingly, strong clustering of individuals whose genotypes were obtained with use of target enrichment, which we attribute to the enrichment bias as described by Davidson et al. [36]. It can also be seen in outgroup $f_3$ heatmap (Fig. S13), where the enriched samples group as a separate cluster (although within the remaining variation) producing a darker-coloured square of higher values. Interestingly however, the clustering of enriched samples is not observed in the MDS plot (Fig. S14) based on the same outgroup $f_3$ statistics that produced highly biased tree and heatmap, nor in the PCA or ADMIXTURE (Fig. 2A and B).

Outgroup-$f_3$ statistics were also estimated for the shotgun sequenced and enriched samples as separate subgroups (MSL.Shotgun.Pop and MSL.Enriched.Pop, respectively; Additional file 2: Table S12A) which showed that both groups show rather similar level of affinities towards test populations. Four of the five closest populations overlap between the two groups (Wielbark Weklice, Late Antiquity Lithuania and Iron Age Sweden, and Iron Age Denmark), although with the enriched group showing slightly higher affinity to Bronze Age Latvia than the shotgun group and shotgun group showing higher affinity to Wielbark Kowalewko than the enriched group.

Neighbour-joining tree constructed on the 1-$f_3$ statistics values using these groupings (i.e., subdivided into MSL.Shotgun.Pop and MSL.Enriched.Pop; Fig. S10), shows identical topology as the corresponding tree using MSL population as single group (tree not shown as it is indeed identical with the MSL.Pop tip in place of the MSL.Shotgun.Pop and MSL.Enriched.Pop clade) and the two subgroups are placed as immediate sister tips in the tree. The differences between $f_3$ with the test populations against Yoruba for the two enrichment-dependent groupings were rather small. The enriched group was, however, estimated to be more affined to some populations (most noticeably to Crimea Pantikapaion and Wielbark Czarnówko) and less to some others (most noticeably to Ukraine Verteba Cave Bell Beaker and Modern Norway) than the shotgun group (Fig. S16).

In $f_4$(MSL.Enriched.Pop, MSL.Shotgun.Pop; Test.Pop, Yoruba) no significant differences were found between the two subgroupings in their affinities to external test populations (Additional file 2: Table S12B)

The bias is further shown by $f_4$ statistics $f_4$(MSL.Shotgun.Pop, MSL.Enriched.Pop/MSL.Pop; MSL.Shotgun.Ind, Yoruba) and $f_4$(MSL.Enriched.Pop/MSL.Pop, MSL.Shotgun.Pop; MSL.Enriched.Ind, Yoruba) (Additional file 2: Table S12C-F). All shotgun-sequenced MSL individuals were significantly closer ($|Z|>3$) to the shotgun-sequenced MSL as a group than to the enriched MSL as a group and than to the whole MSL as a group or any other tested population. Correspondingly, all enriched MSL individuals were significantly closer ($|Z|>3$) to the enriched MSL as a group than to the shotgun-sequenced MSL as a group and than to the whole MSL as a group. There were, however, shotgun samples that were closer to some test populations than to the enriched MSL subgroup and enriched samples that were closer to some test populations than to the shotgun MSL subgroup. This confirms the presence of bias as described by Davidson et al. [36], which needs to be considered while interpreting the obtained results.

The profoundness of the bias was further explored using additional set of $f_4$ statistics. The individuals within the two subgroups are generally homogenously affined to their respective subgroups with only 9% $f_4$(Yoruba, MSL.Shotgun.Pop; MSL.Shotgun.Ind1, MSL.Shotgun.Ind2) and 7% of $f_4$(Yoruba, MSL.Enriched.Pop; MSL.Enriched.Ind1, MSL.Enriched.Ind2) statistics with $|Z|>3$ (Additional file 2: Table S12G and H, respectively). Shotgun sequenced individuals were in 99.5% of tests significantly closer to MSL.Shotgun.Pop than to any reference population (the rest yielding a $|Z|<3$ estimate rather than significant affinity to the reference population) and all enriched were significantly closer to MSL.Enriched.Pop than to any reference population (Additional file 2: Table S12I and J, respectively).

Furthermore, while all the MSL individuals are significantly closer to the MSL.Pop as a whole than to any reference population (Additional file 2: Table S15), only in case of around 50% of the $f_4$(MSL.Enriched.Pop, Test.Pop, MSL.Shotgun.Ind, Yoruba) and $f_4$(MSL.Shotgun.Pop, Test.Pop, MSL.Enriched.Ind, Yoruba) statistics yielded $Z>3$, i.e. only around a half of the tests showed the individuals to be significantly closer to the non-respective MSL subgroup than to non-MSL populations (Additional file 2: Table S12K and L). This result clearly shows that the division into the two subgroups within the MSL group is very strong.

To test for the effect of the enrichment bias on the relatedness estimations, we performed t-tests for BREADR pmr, READ kinship coefficient and NGSrelate rab values between pairs of differently sequenced vs between pairs of enriched individuals, and between pairs of shotgun sequenced vs between pairs of enriched individuals. The BREADR pmr were higher in enriched-enriched pairs that in pairs of varying sequencing strategy with $p=0.026$ and than in shotgun-shotgun pairs with $p=0.053$. READ kinship coefficients differed with, respectively, $p=0.008$ and $p=0.001$, and NGSrelate theta with $p=0.373$ and $p=0.94$. It is therefore possible that the bias may affect kinship metrics, at least in some cases.

The presence of enrichment bias has been strongly supported by various statistics. Although we cannot exclude the possibility that the clustering of enriched samples separately from the shotgun sequenced may be driven by true higher affinity between the individuals whose samples happened to have been subjected to enrichment, we apply additional caution and consideration for this bias when interpreting the results of our population analysis.

## Supplementary Figures S4-S16

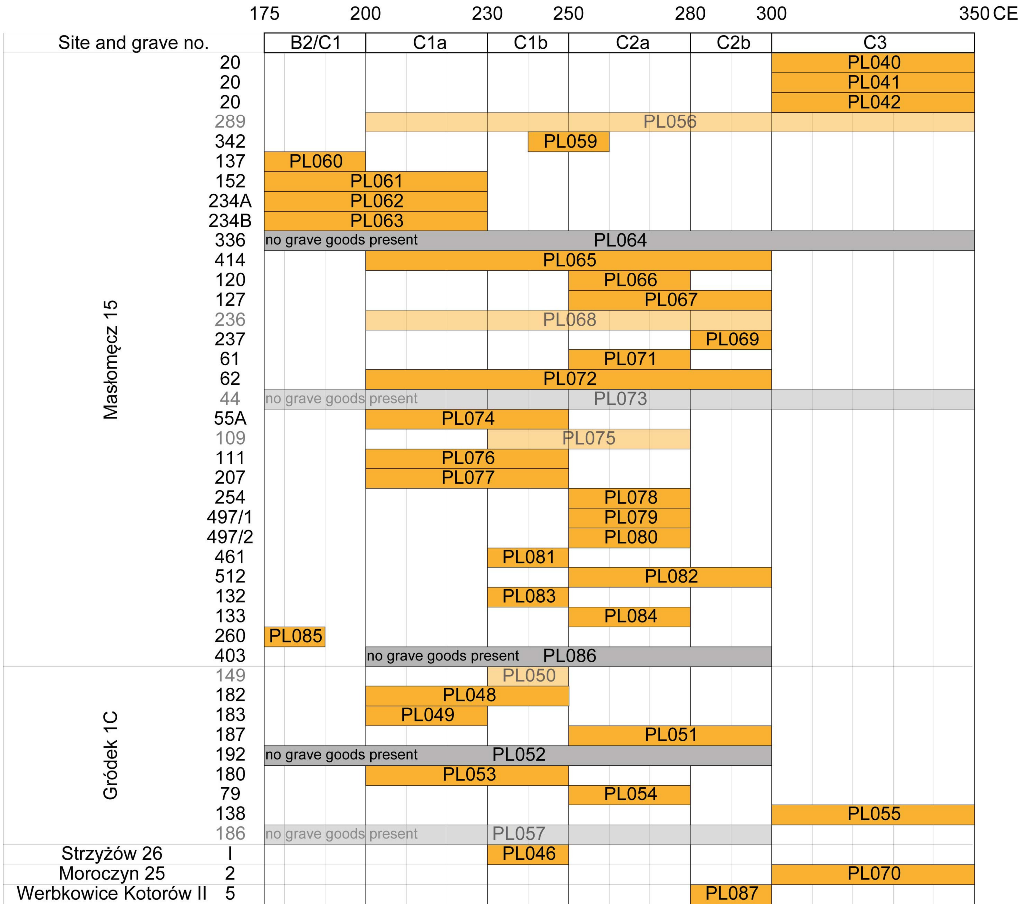


Fig. S4. Dating of the analysed skeletal remains based on the archaeological context. Orange shading indicates period to which given individual was assigned to, based on the associated grave goods. Archaeological site and grave number for each individual is provided on the left side. Samples not included in the analyses due to insufficient DNA preservation or contamination were only lightly shaded. Period labels and age ranges (year CE) are provided in the two top rows of the table.

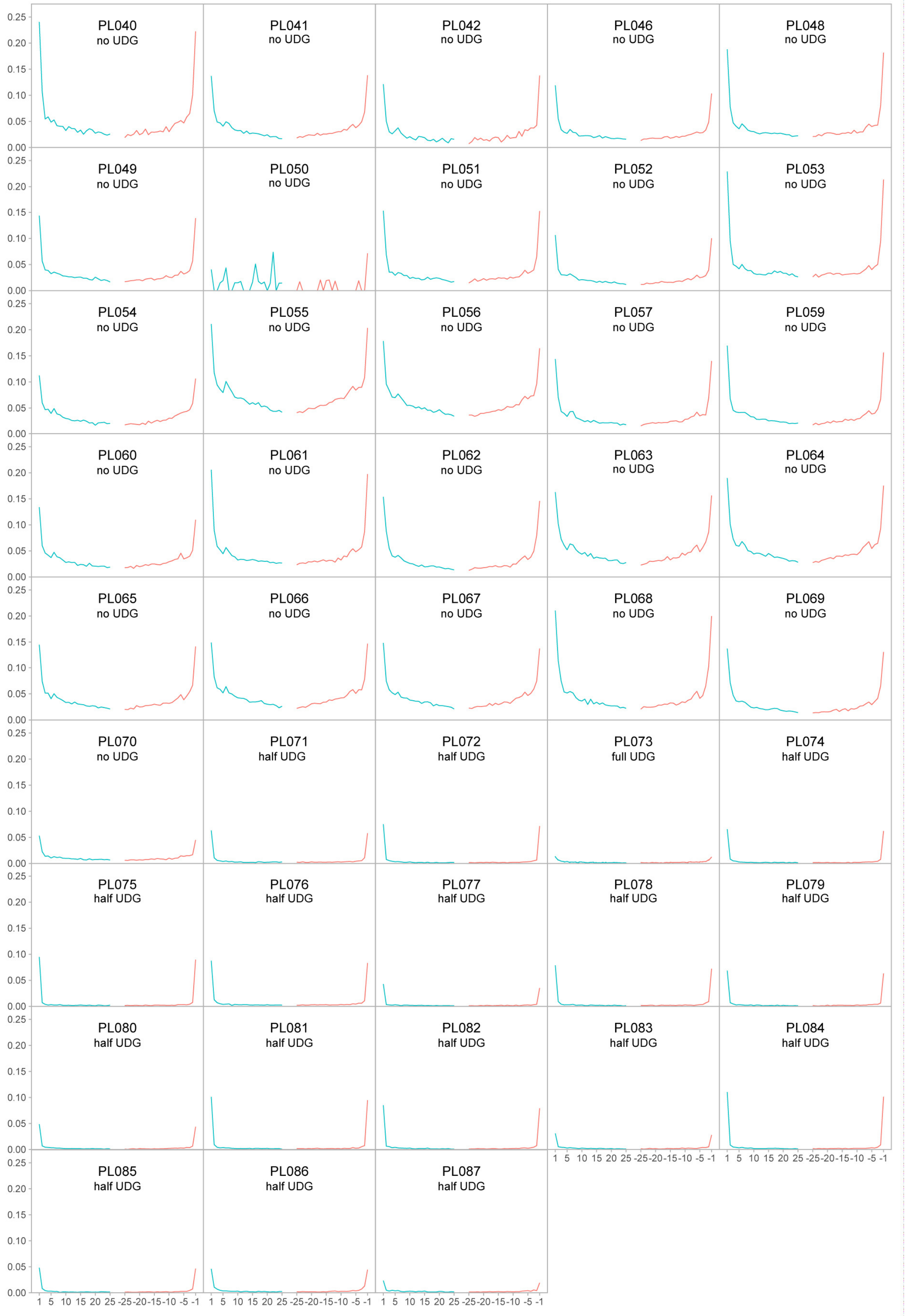


Fig. S5. DNA damage patterns on read termini resulting from cytosine deamination. The UDG treatment used for each library is marked below the individual label; the libraries were either not treated with UDG (no UDG), treated with UDG for one hour (half UDG), or for three hours (full UDG).

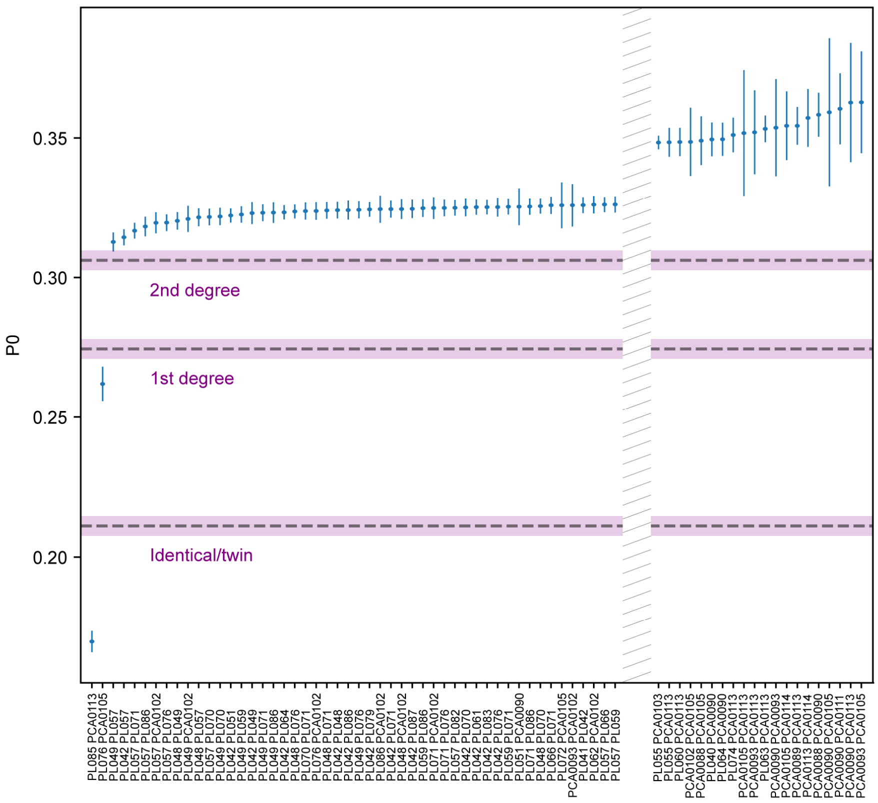


Fig. S6. Estimation of relatedness using READ v2.0. Only pairs with lowest and highest P0 values were retained in the figure for readability.

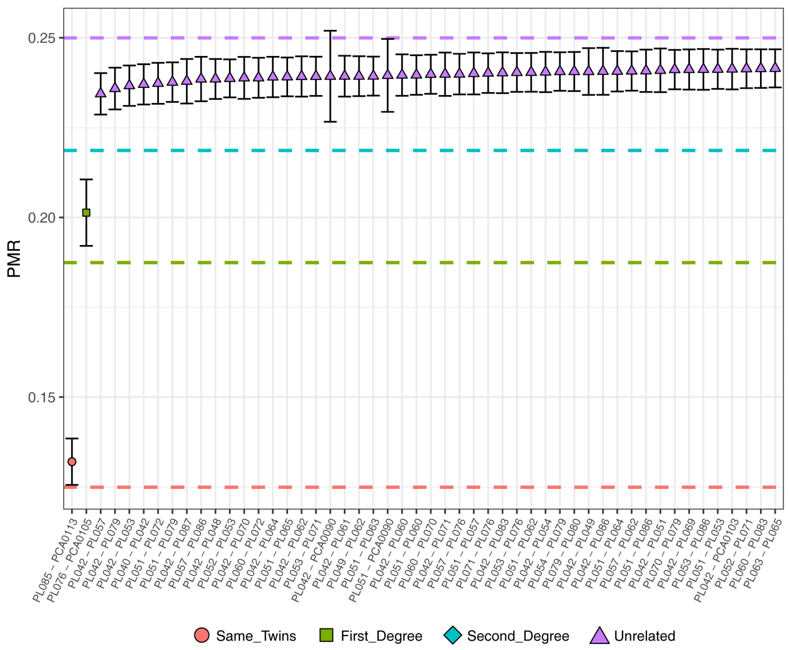


Fig. S7. Estimation of relatedness using BREADR.

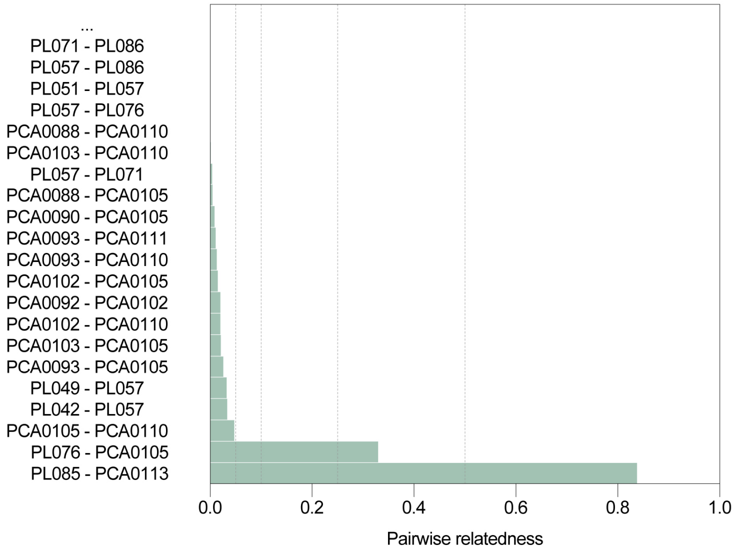


Fig. S8. Estimation of relatedness using NGSrelate.

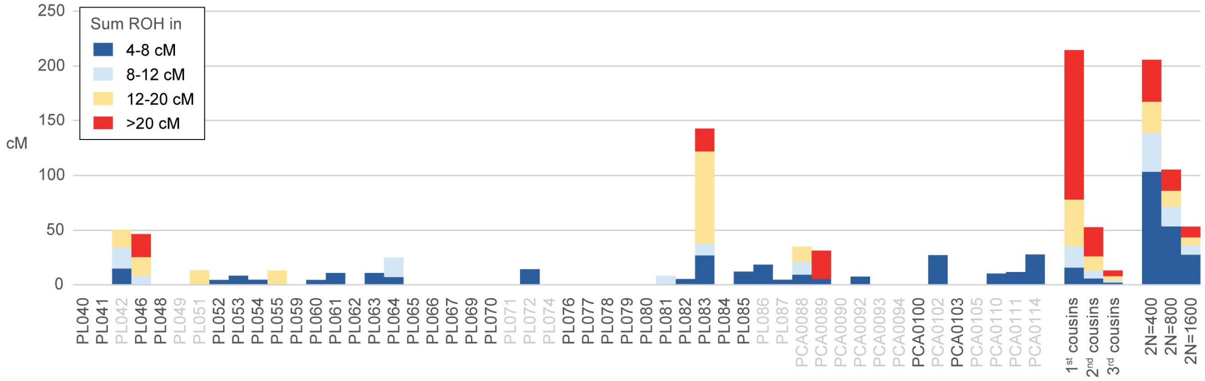


Fig. S9. ROH segments length among the MSL individuals. Individuals with less than 500k “1240k” SNPs genotyped (with higher probability of false ROH detection) are labelled in grey. On the right, values expected for the close-kin parents and low effective population size [34].

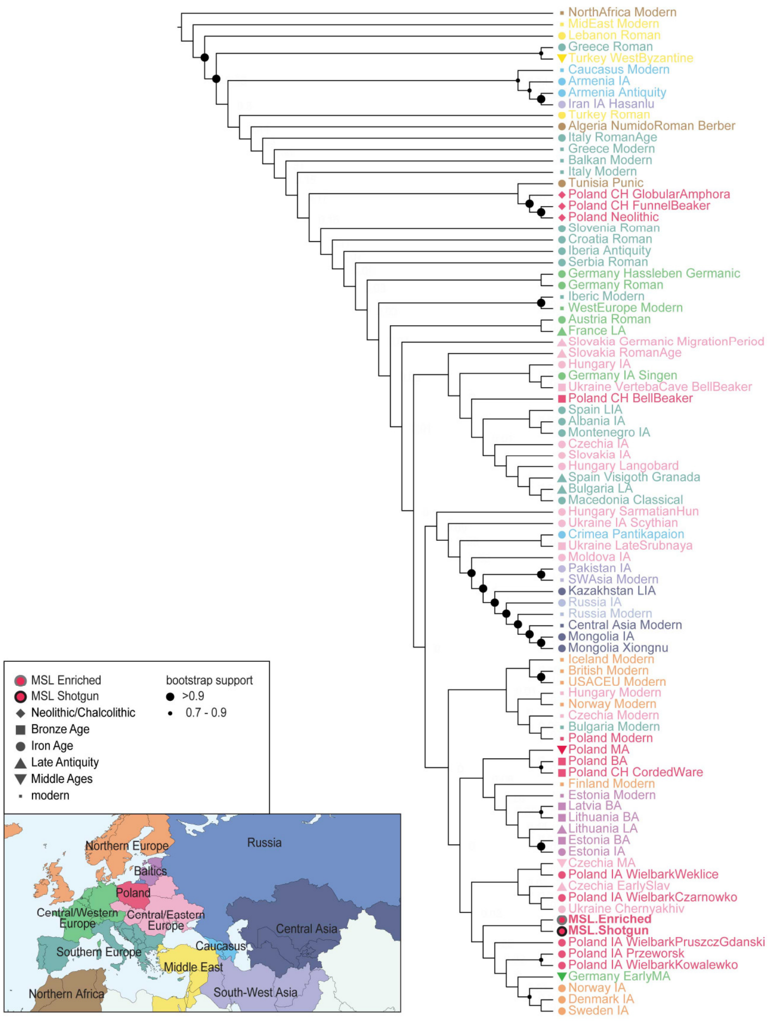


Fig. S10. Neighbour-joining tree (cladogram) based on outgroup $f_3$-statistics [1-$f_3$ (population/individual1, population/individual2; Yoruba)] with the MSL population split into two groups: shotgun-sequenced (MSL.Shotgun) and enriched using Human Affinities Prime Kit (Arbor Bioscientific; MSL.Enriched).

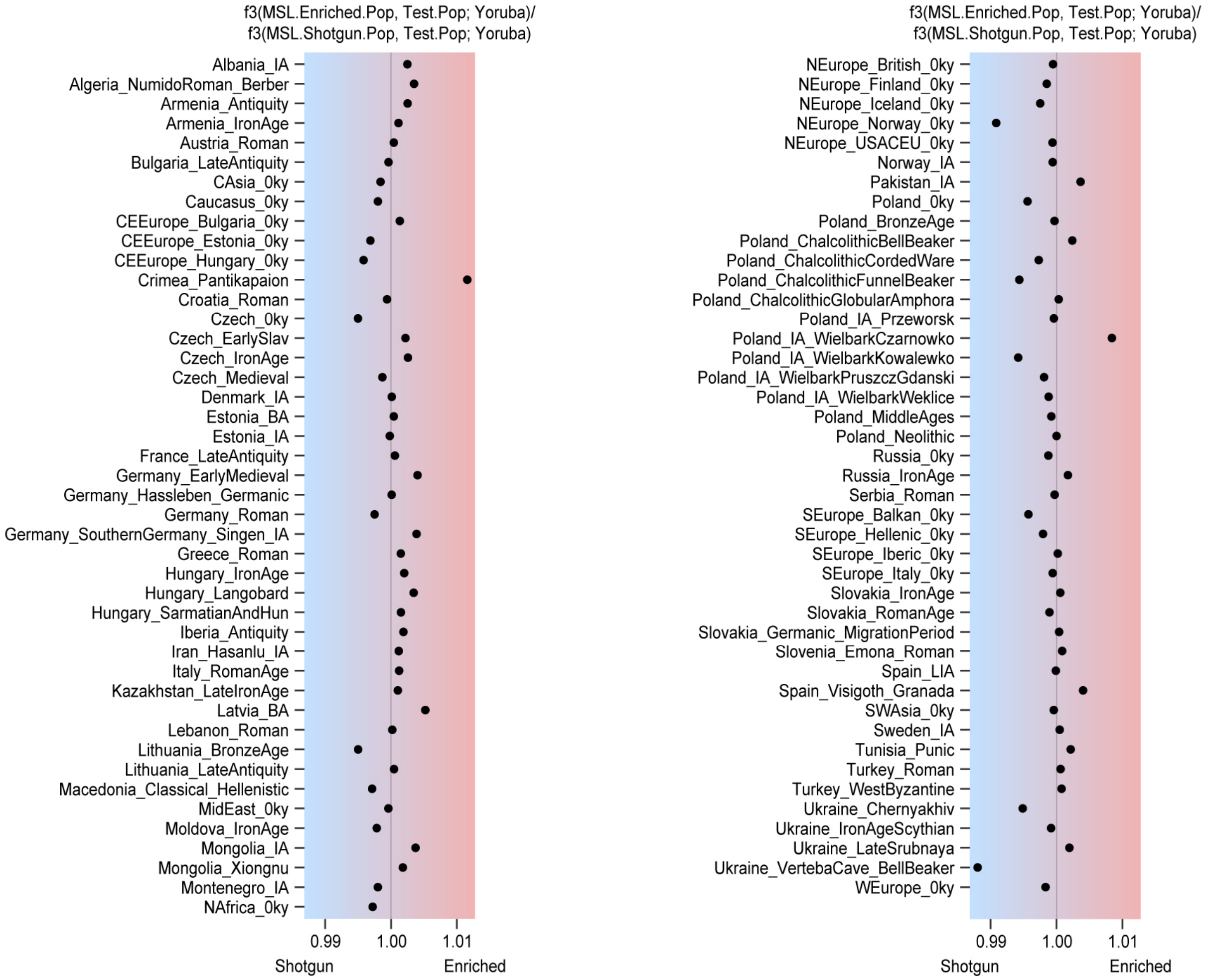


Fig. S11. Relative affinities of the enriched- and shotgun-generated individuals as groups to test populations. Each black circle marker represents a ratio of point estimate values ($f_3$(MSL.Enriched.Pop, Test.Pop; Yoruba)/$f_3$(MSL.Shotgun.Pop, Test.Pop; Yoruba)) for test populations labelled on the left.

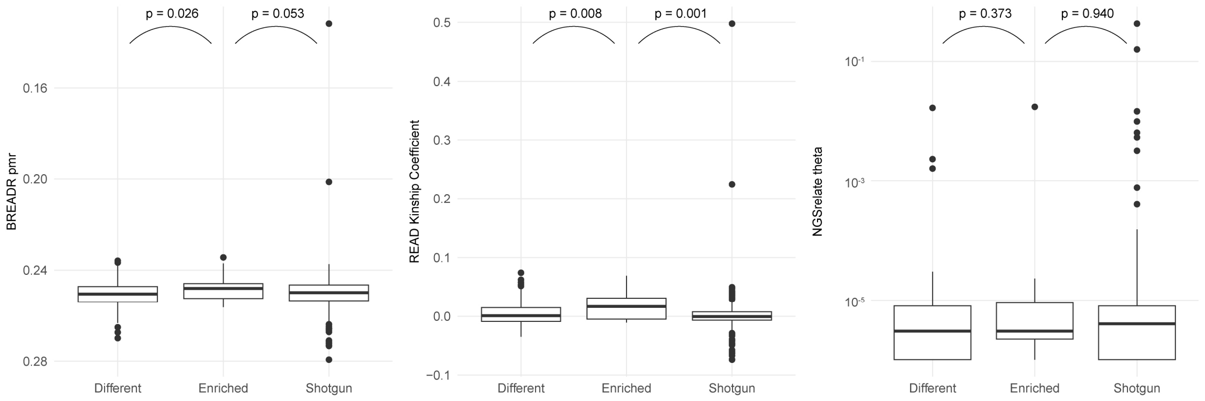


Fig. S12. The effect of enrichment treatment on the kinship metrics. P-values from two-sided Welch two sample T-test between the given metrics in enriched-shotgun (“Different”) pairs and enriched-enriched pairs and between enriched-enriched and shotgun-shotgun pairs. Note that BREADR pmr value axis is reversed to visually represent the level of closeness, not distance, in accordance with the remaining shown metrics.

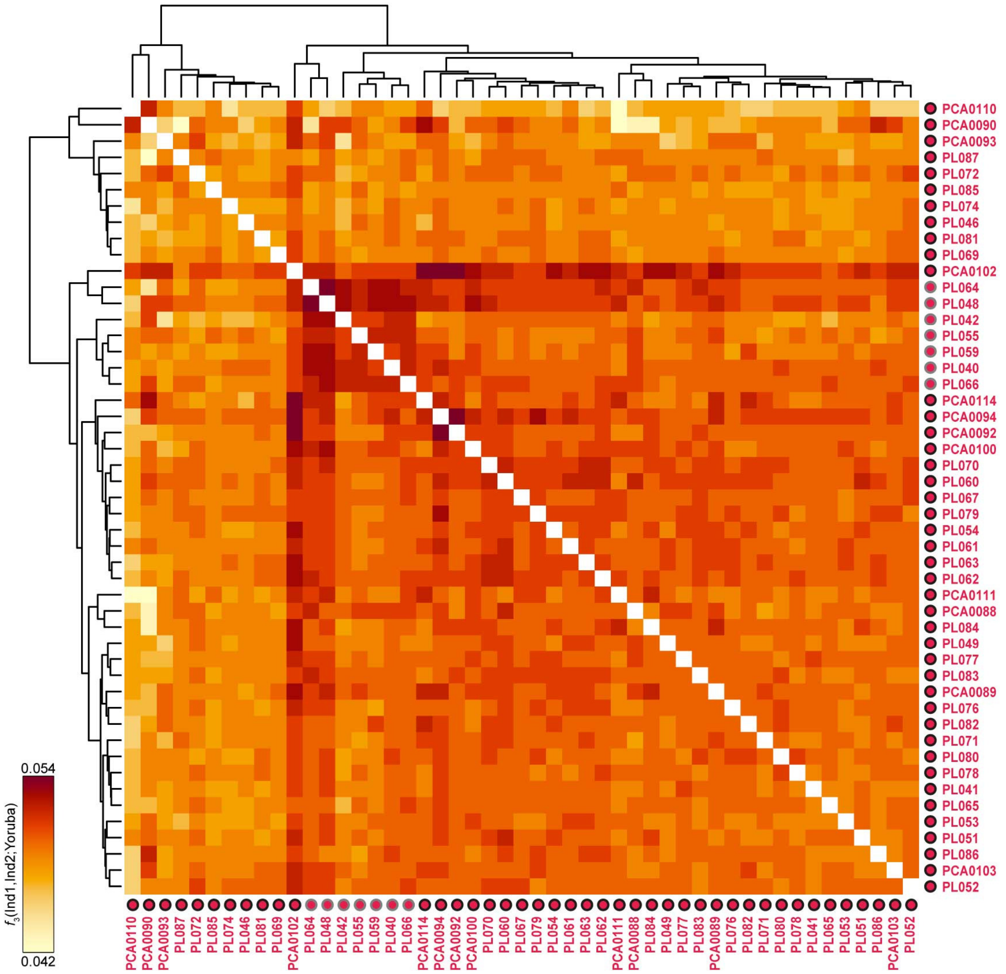


Fig. S13. Outgroup $f_3$ heatmap for the MSL individuals. Individual PCA0105, a first degree relative of PL076, was excluded from the plot as they distort the $f_3$ distance variation in the dataset. Markers for the enrichment-derived samples are outlined in grey.

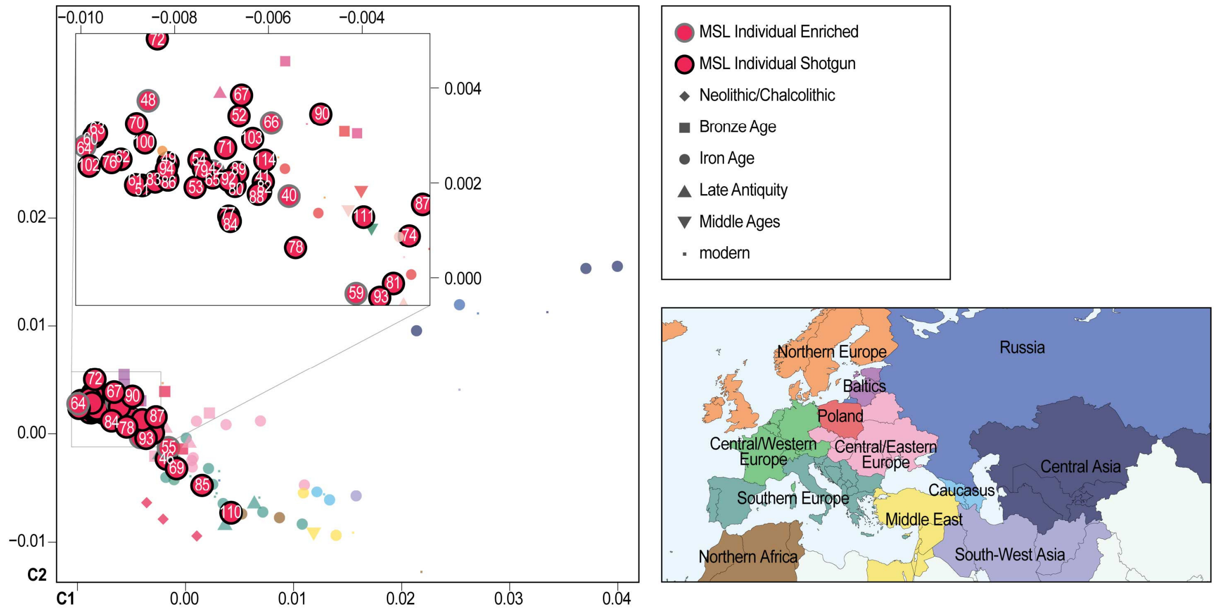


Fig. S14. MDS plot for the outgroup $f_3$-statistic [1- $f_3$(individual/population, Test, Yoruba)] matrix and a zoomed-in high-density portion of the plot. Individual labels were shortened for visibility to the two or three terminal digits. The map shows regional groupings and colour-scheme assigned to the individuals' skeletal remains' countries of origin.

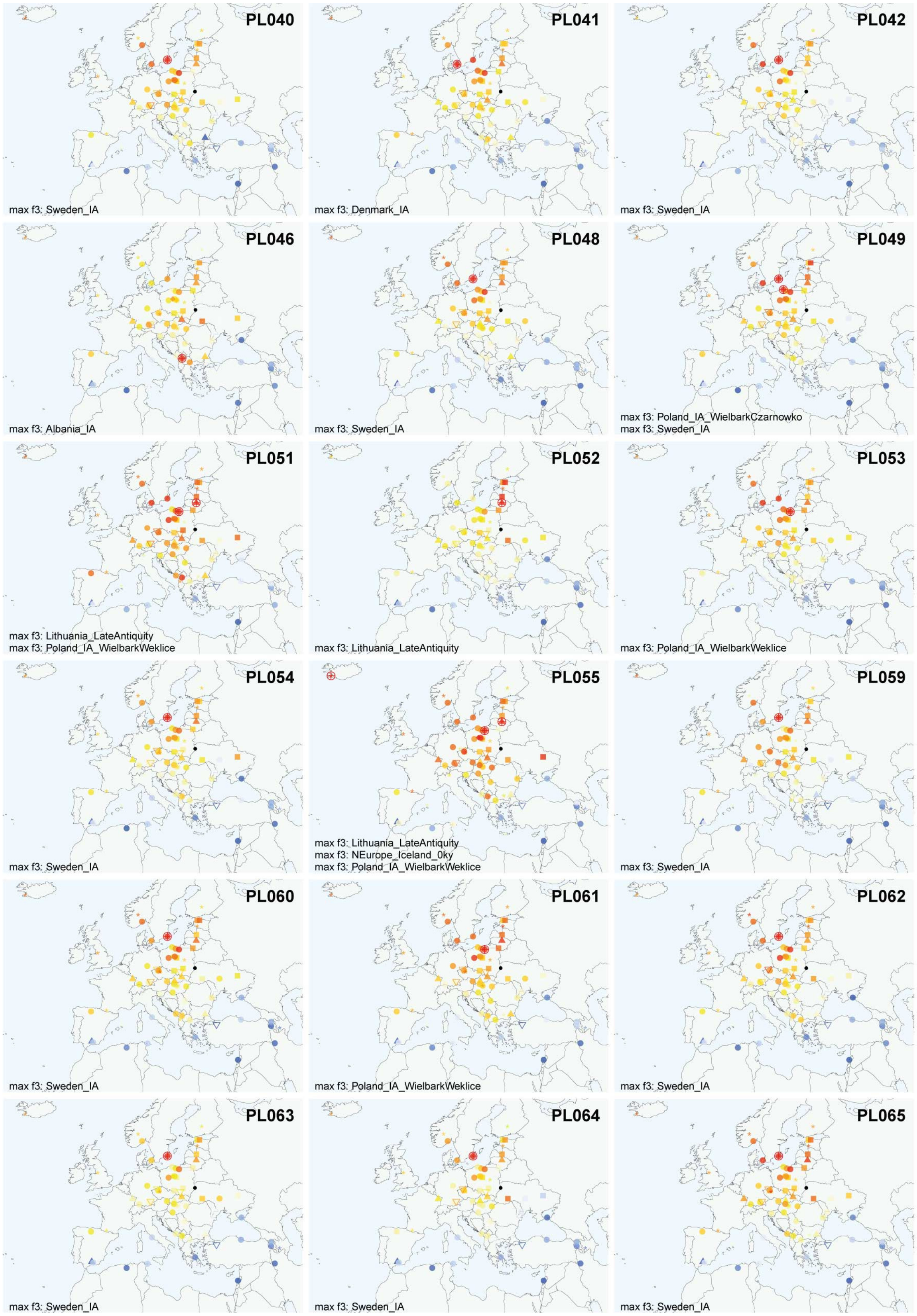
PL040
max f3: Sweden_IA
PL041
max f3: Denmark_IA
PL042
max f3: Sweden_IA
PL046
max f3: Albania_IA
PL048
max f3: Sweden_IA
PL049
max f3: Poland_IA_WielbarkCzarnowko
max f3: Sweden_IA
PL051
max f3: Lithuania_LateAntiquity
max f3: Poland_IA_WielbarkWeklice
PL052
max f3: Lithuania_LateAntiquity
PL053
max f3: Poland_IA_WielbarkWeklice
PL054
max f3: Sweden_IA
PL055
max f3: Lithuania_LateAntiquity
max f3: NEurope_Iceland_0ky
max f3: Poland_IA_WielbarkWeklice
PL059
max f3: Sweden_IA
PL060
max f3: Sweden_IA
PL061
max f3: Poland_IA_WielbarkWeklice
PL062
max f3: Sweden_IA
PL063
max f3: Sweden_IA
PL064
max f3: Sweden_IA
PL065
max f3: Sweden_IA

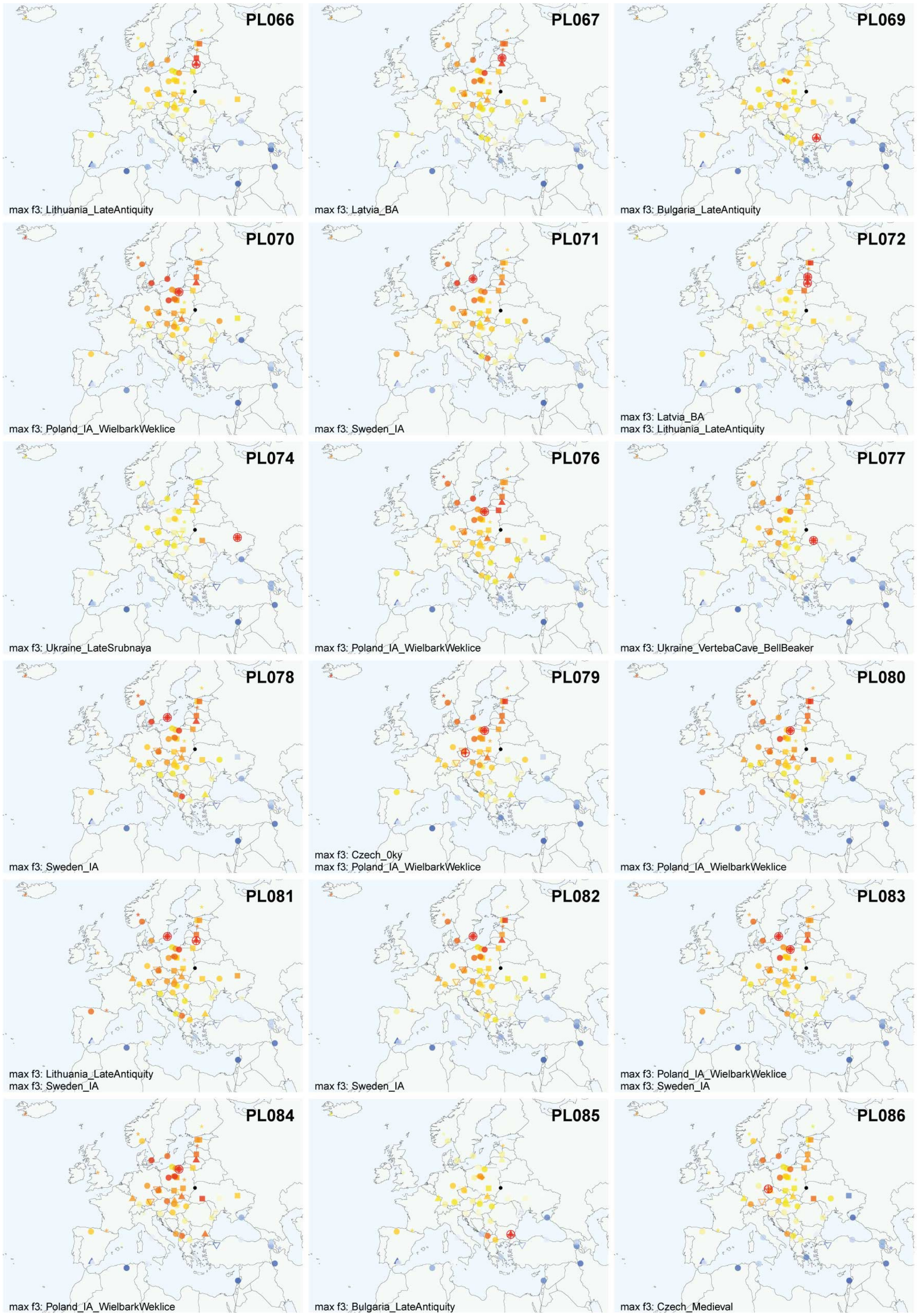
PL066
max f3: Lithuania_LateAntiquity
PL067
max f3: Latvia_BA
PL069
max f3: Bulgaria_LateAntiquity
PL070
max f3: Poland_IA_WielbarkWeklice
PL071
max f3: Sweden_IA
PL072
max f3: Latvia_BA
max f3: Lithuania_LateAntiquity
PL074
max f3: Ukraine_LateSrubnaya
PL076
max f3: Poland_IA_WielbarkWeklice
PL077
max f3: Ukraine_VertebaCave_BellBeaker
PL078
max f3: Sweden_IA
PL079
max f3: Czech_0ky
max f3: Poland_IA_WielbarkWeklice
PL080
max f3: Poland_IA_WielbarkWeklice
PL081
max f3: Lithuania_LateAntiquity
max f3: Sweden_IA
PL082
max f3: Sweden_IA
PL083
max f3: Poland_IA_WielbarkWeklice
max f3: Sweden_IA
PL084
max f3: Poland_IA_WielbarkWeklice
PL085
max f3: Bulgaria_LateAntiquity
PL086
max f3: Czech_Medieval

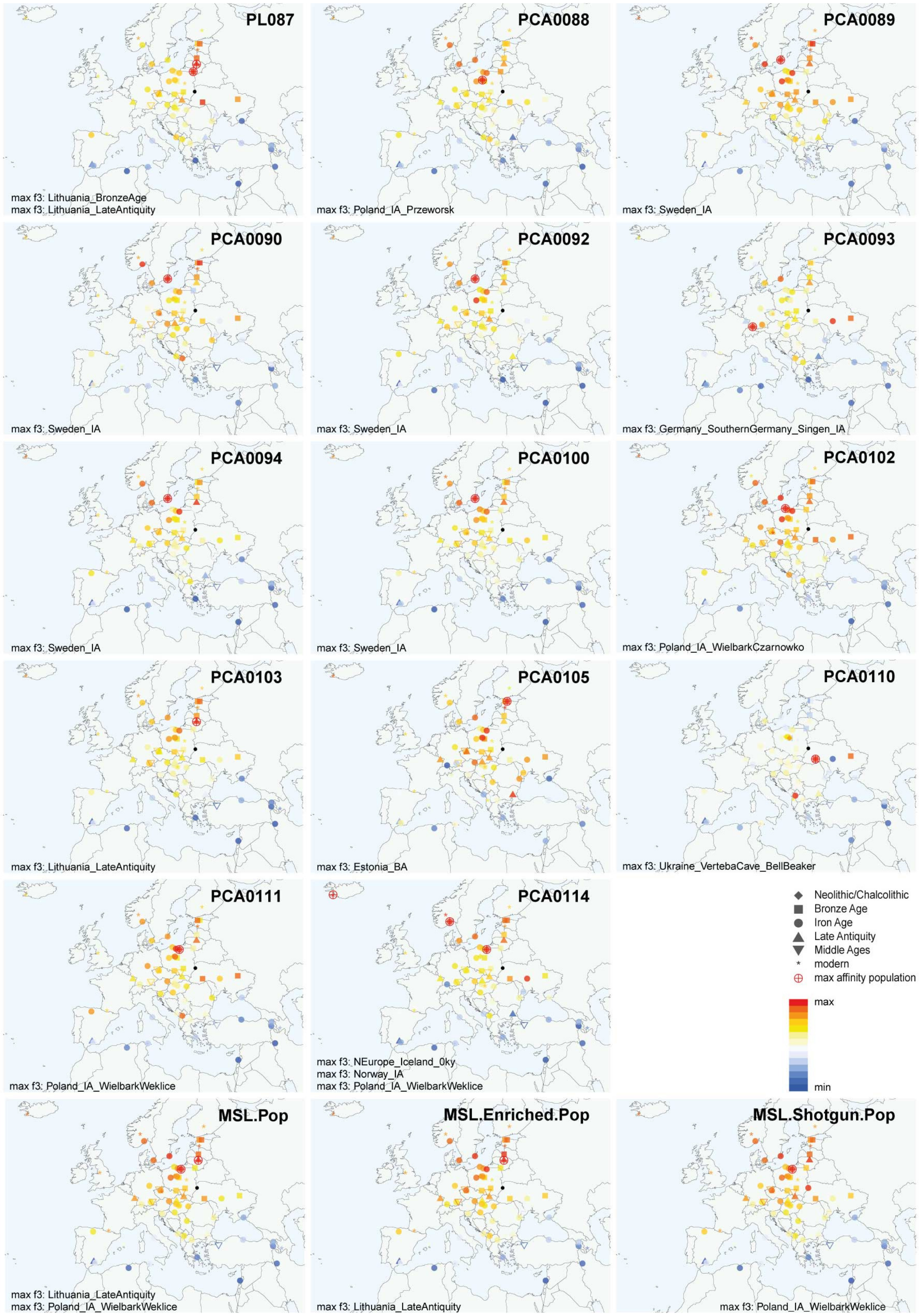


Fig. S15. Distribution of outgroup-$f_3$-based genetic affinity to the reference populations (excluding MSL.Pop – which has the highest f3 statistics value among all tested populations for all the individuals – and NAfrica_0ky, Mongolia_Xiongnu, Mongolia_IA, MidEast_0ky, SWAsia_0ky, Pakistan_IA, CAsia_0ky, Russia_0ky, Russia_IronAge, Kazakhstan_LateIronAge – for which no notable affinities were found among the studied individuals but which are geographically distant so would require smaller scale i.e. less readable maps – for enhanced visibility of variation in affinity levels).

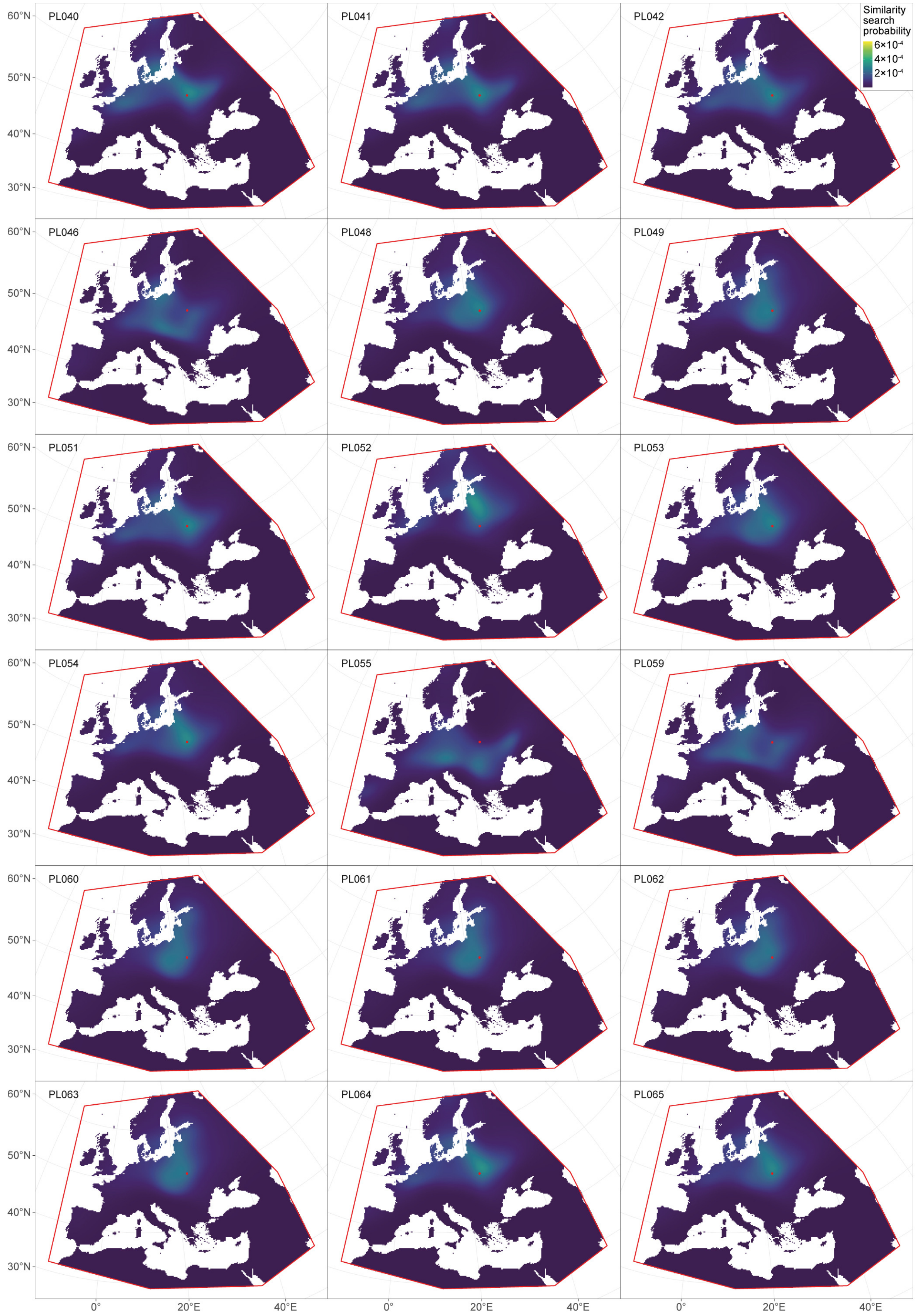
PL040
PL041
PL042
Similarity search probability
6×10⁻⁴
4×10⁻⁴
2×10⁻⁴
PL046
PL048
PL049
PL051
PL052
PL053
PL054
PL055
PL059
PL060
PL061
PL062
PL063
PL064
PL065
60°N
50°N
40°N
30°N
0°
20°E
40°E

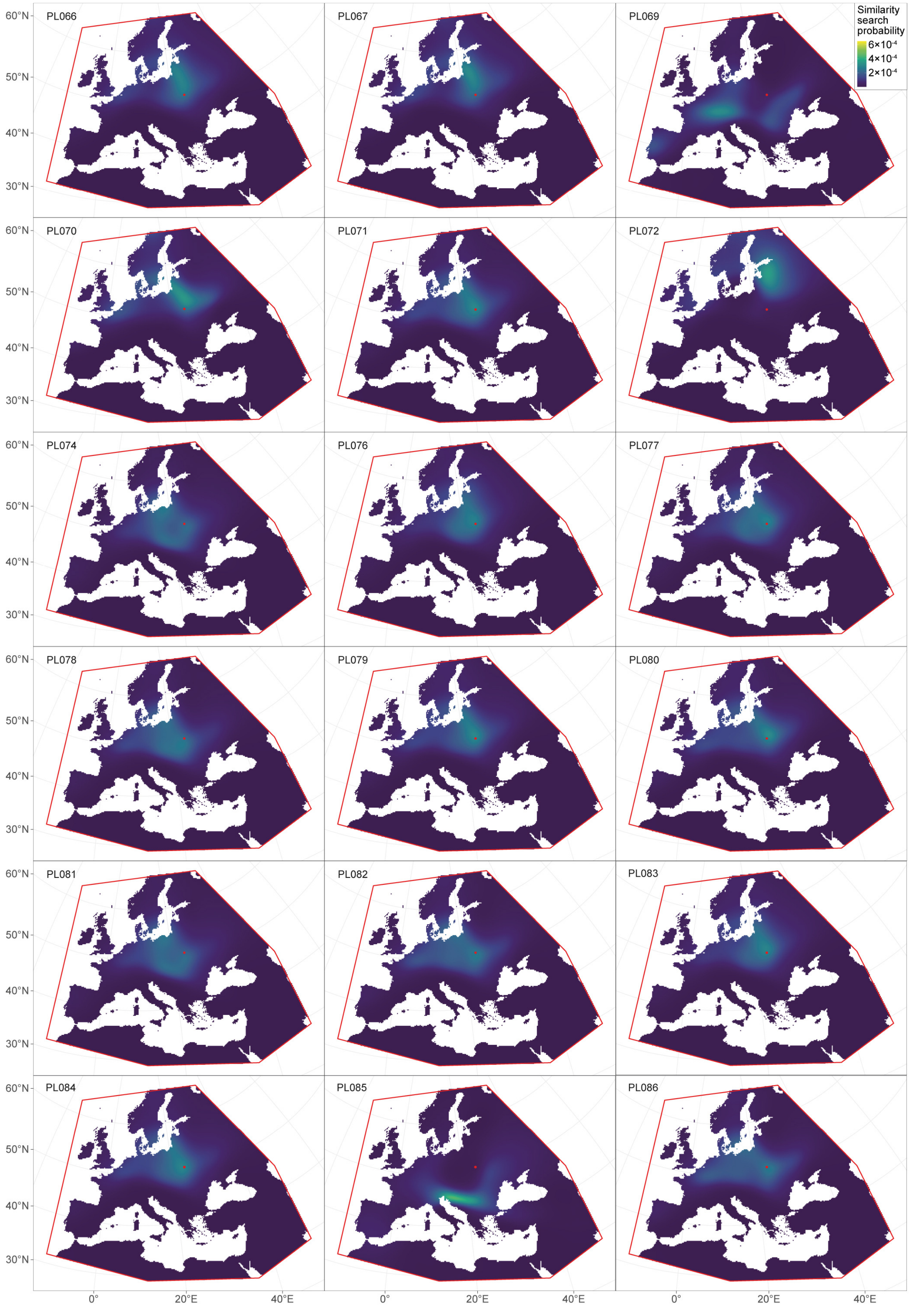
PL066
PL067
PL069
Similarity search probability
6×10⁻⁴
4×10⁻⁴
2×10⁻⁴
PL070
PL071
PL072
PL074
PL076
PL077
PL078
PL079
PL080
PL081
PL082
PL083
PL084
PL085
PL086
60°N
50°N
40°N
30°N
0°
20°E
40°E

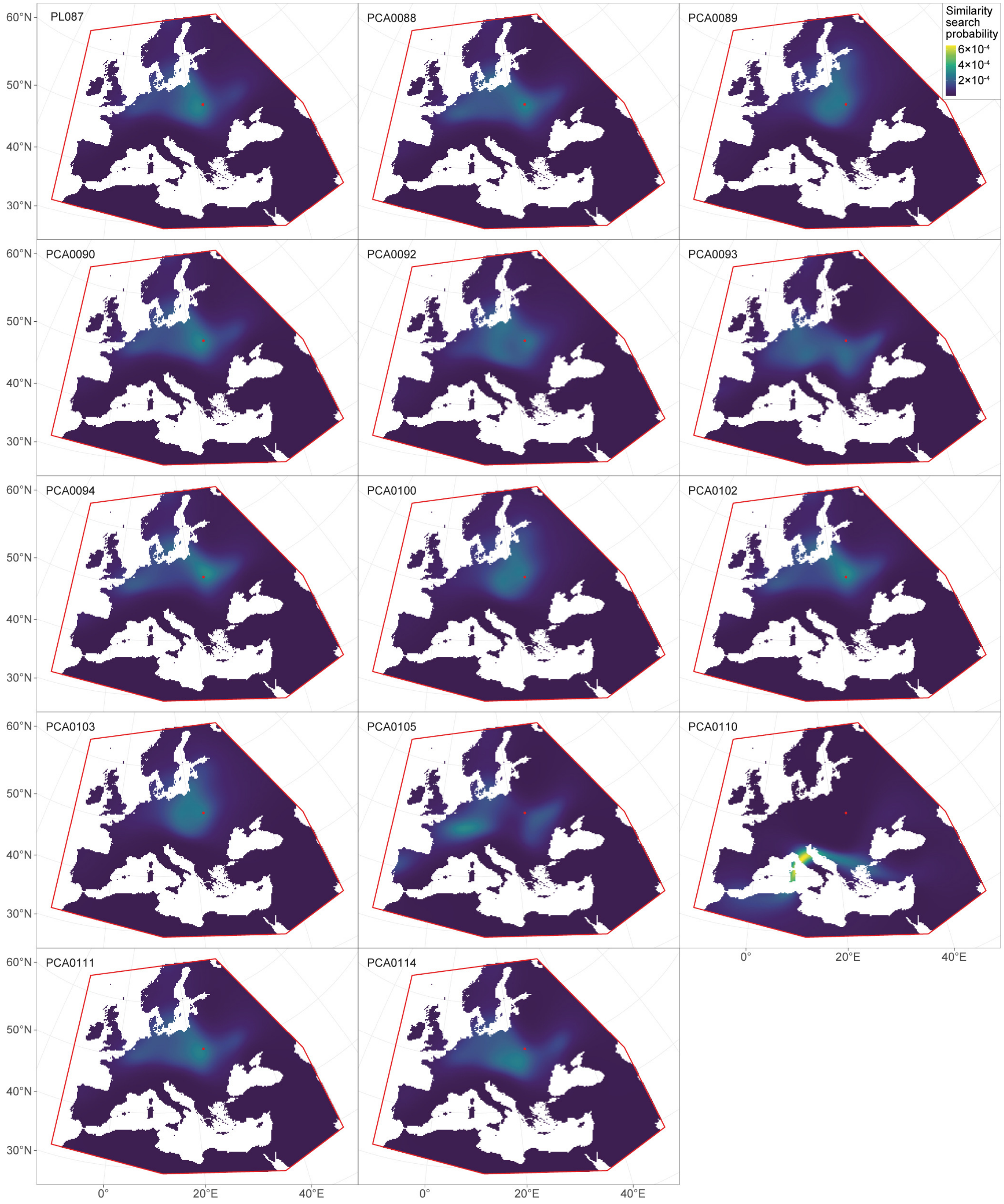


Fig. S16. Mobest analysis of geographic distribution of each individual's genomic ancestry (based on two first PCA coordinates). Red dot marks the origin place of the individual's skeletal remains. Coloured heatmap shows the distribution of the individual's ancestry across Europe at the time of the archaeologically estimated time of death.
